# Nigra‐Subthalamic Dopaminergic Circuitry Modulates and Represents Distinct Pain Modality in Physiological and Pain States in Mice

**DOI:** 10.1002/advs.202519913

**Published:** 2026-04-07

**Authors:** Ying Ji, Shuyi Li, Jiaqi Zhang, Xiang‐Ying Xu, Cui Yin, Mu Niu, Danyang Liu, Yanyu Jiang, Yong Niu, Su Liu, Bin Zhang, Guiyun Cui, Chunyi Zhou, Cheng Xiao

**Affiliations:** ^1^ Jiangsu Province Key Laboratory of Anesthesiology School of Anesthesiology Xuzhou Medical University Xuzhou Jiangsu China; ^2^ Jiangsu Province Key Laboratory of Anesthesiology and Brain Science Xuzhou Medical University Xuzhou Jiangsu China; ^3^ Department of Anesthesiology Xuzhou Central Hospital Xuzhou Jiangsu China; ^4^ Department of Anesthesiology Affiliated Hospital of Xuzhou Medical University Xuzhou Jiangsu China; ^5^ Department of Neurology Affiliated Hospital of Xuzhou Medical University Xuzhou Jiangsu China; ^6^ Key Laboratory of Chemical Safety and Health, National Institute for Occupational Health and Poison Control Chinese Center for Disease Control and Prevention Beijing China; ^7^ Department of General Surgery The Affiliated Hospital of Xuzhou Medical University Xuzhou Jiangsu China

**Keywords:** dopamine receptors, dopaminergic neurons, neuropathic pain, Parkinsonian pain, substantia nigra pars compacta, subthalamic nucleus

## Abstract

Dopaminergic (DA) neurons in the substantia nigra pars compacta (SNc) degenerate in Parkinson's disease (PD). Although pain is a common non‐motor symptom in PD, it remains unclear whether and how degeneration of SNc DA neurons contributes to hyperalgesia. In the present study, we revealed a nigro‐subthalamic DA circuit, composed of a subset of SNc DA neurons, the SNc DA projection to the subthalamic nucleus (STN), and the downstream STN neurons. These components regulate mechanical, but not thermal, pain threshold on the contralateral side, exhibiting distinct responses to mechanical and thermal stimuli which varied in neuropathic pain and Parkinsonian mice. D2‐, but not D1‐like, dopamine receptors in the STN were involved in these processes, and their activation mitigated mechanical hyperalgesia in both neuropathic pain and Parkinsonian mice. The GABAergic neurons in the substantia nigra pars reticulata (SNr) responded to pain stimulation and facilitated pain responses in SNc DA neurons. Thus, the SNr^GABA^‐SNc^DA^‐STN pathway is involved in the modulation and processing of pain in both physiological and chronic pain states and may be a potential therapeutic target for both neuropathic and Parkinsonian pain.

## Graphical abstract

Nigral dopaminergic (DA) neurons modulate and represent pain with a preference to a particular modality (mechanical) and laterality (contralateral), which are controlled by nigral GABAergic neurons. The pain modulation is mimicked by the nigro‐subthalamic projection and its downstream neurons, involving D2‐like receptors. Activation of the nigro‐subthalamic projection or subthalamic D2‐like receptors mitigates neuropathic pain and Parkinsonian pain.

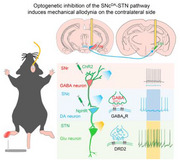

## 1 Introduction

Parkinson's disease (PD), as one of the leading neurodegenerative diseases, affects 1%–2% of the senior population. Besides debilitating motor symptoms, pain is commonly reported in PD patients, with a prevalence between 40%–85% [1, 2, 3]. In some PD patients, pain develops even earlier than motor symptoms, worsening as the disease progresses, and is usually more severe compared to non‐PD pain [4, 5]. Although pain ranks among the top ten symptoms affecting the quality of life of PD patients in both early and late stages [6], its treatment is often secondary to that of motor symptoms, benefiting only 30% of patients [3, 7]. To address this gap, further investigations are needed to explore the neural circuit mechanisms underlying PD‐related pain.

Degeneration of dopaminergic (DA) neurons in the substantia nigra pars compacta (SNc) is a key pathological feature in PD. Accumulating evidence suggests that the death of SNc DA neurons may be a major contributor to PD‐related pain. For instance, animal models established by selective lesion of SNc DA neurons exhibit mechanical and thermal hyperalgesia and exacerbation of inflammatory and visceral pain [1, 8, 9]. Additionally, loss‐of‐function polymorphisms in type 3 dopamine receptors (DRD3, rs6280) and type 2 dopamine receptors (DRD2, rs2283265 and rs1076560) are significant risk factors for PD pain [10]. Blocking D2‐like dopamine receptors impairs the adaptation mechanism to repetitive pain stimulation and exacerbates pain sensation [11], while levodopa reduces levels of pain and associated discomfort [12, 13, 14, 15]. Furthermore, rodent studies indicate that electrical stimulation of the tail inhibits the majority of SNc neurons in rats [16], and intracranial electrical stimulation of the SNc attenuates responses of deep dorsal horn neurons in the lumbar spinal cord to nociceptive mechanical and electrical stimulation on the gastrocnemius in cats [17]. These findings suggest that dysfunction of the dopaminergic system, including that originating from SNc DA neurons, may play a significant role in Parkinsonian pain. However, cell‐ and projection‐specific approaches are necessary to delineate the neural circuits through which SNc DA neurons are implicated in these events.

The subthalamic nucleus (STN), primarily composed of glutamatergic neurons, is regulated by the DA system [15, 18, 19]. In PD, the STN exhibits pathophysiological abnormalities, including hyper‐excitability, irregular neuronal firing patterns, and pathological beta oscillation [8, 15, 19, 20]. Deep‐brain stimulation of the STN effectively controls motor symptoms in PD patients and unintentionally attenuates pain [15, 21, 22]. These studies suggest a role for STN pathophysiology in PD‐related pain. Our previous studies have demonstrated that optogenetic stimulation of STN neurons decreases pain threshold in naïve mice. In contrast, optogenetic inhibition of STN neurons alleviates hyperalgesia in Parkinsonian, inflammatory, and neuropathic pain [8, 23, 24]. This may indicate that hyperactivity in STN neurons is a sufficient and necessary condition for these pain states. However, it remains unknown whether and how STN neurons involved in pain modulation are controlled by SNc DA neurons.

In this study, we uncover a neural circuitry within the basal ganglia—the substantia nigra pars reticulata (SNr)‐SNc‐STN pathway—that is involved in the modulation and signaling of pain. SNr GABAergic neurons, SNc DA neurons, and the SNc‐STN projection display distinct pain response patterns based on the modality and laterality of the stimulation under both physiological and pathophysiological conditions. Additionally, D2‐like dopamine receptors in the STN are part of this pain modulation pathway and may serve as potential targets for effectively alleviating mechanical hyperalgesia in inflammatory, neuropathic, and Parkinsonian pain. This study offers new insights into the role of basal ganglion circuits in modulating pain under physiological and pathological conditions.

## 2 Results

### 2.1 The SNc‐STN DA Projection Mediates the Modulation of Mechanical Threshold by SNc DA Neurons

**FIGURE 1:**
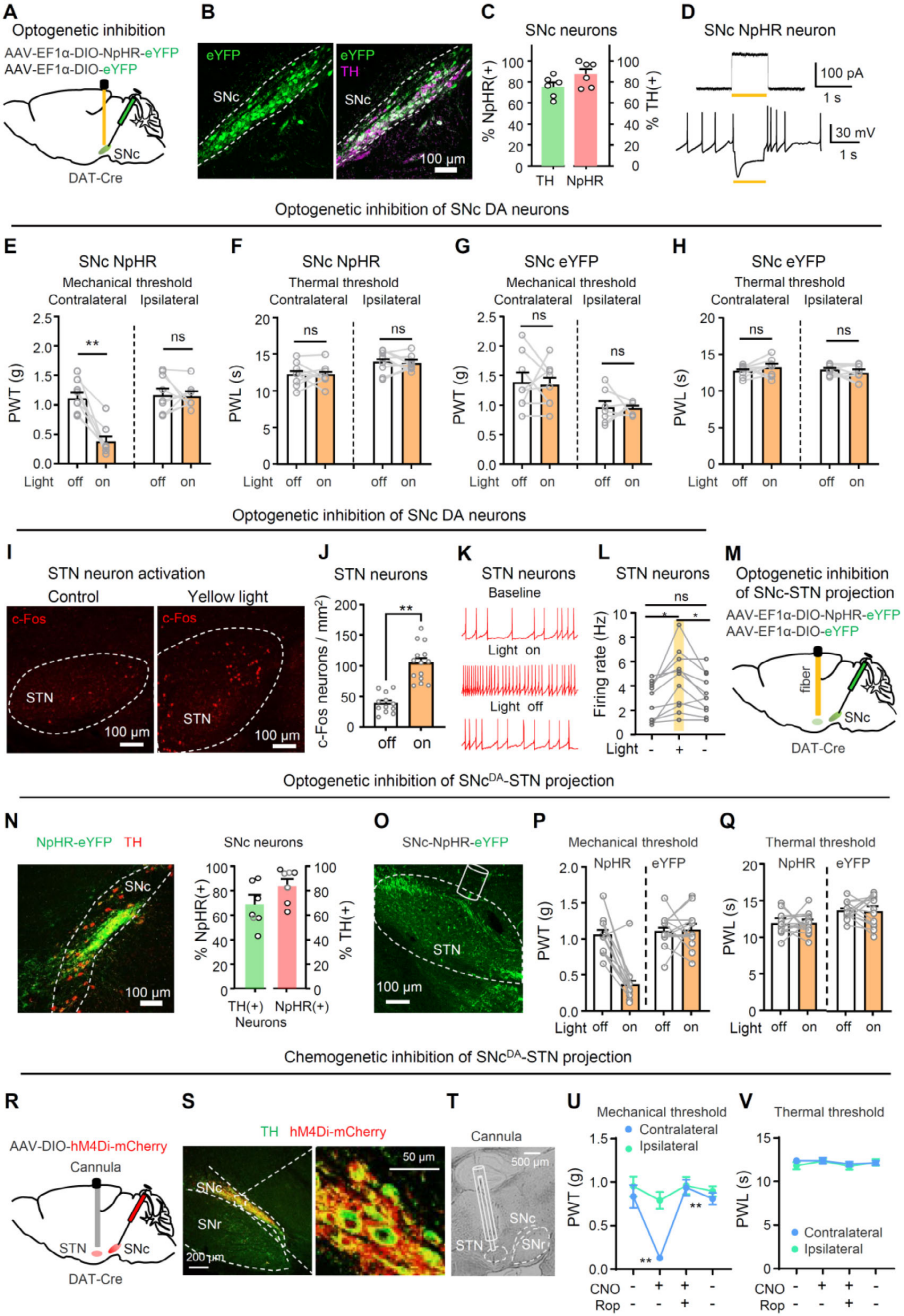
Inhibition of SNc DA neurons and the SNc‐STN DA projection causes mechanical allodynia on the contralateral side. (A–C) Schematic diagram (A), representative images (B), and summary of efficiency (green bar) and specificity (red bar) (C) for transfection of NpHR or eYFP into SNc DA neurons. (D) Representative traces showing that yellow light illumination evoked an outward current (upper panel) and inhibited firing (lower panel) in an NpHR‐eYFP‐expressing SNc neuron. (E–H) Effects of optogenetic inhibition of SNc DA neurons on mechanical paw withdrawal threshold (PWT) and thermal paw withdrawal latency (PWL) on either side of DAT‐Cre mice subjected to injection of AAV‐EF1α‐DIO‐NpHR‐eYFP (NpHR, *n* = 9) or AAV‐EF1α‐DIO‐eYFP (eYFP, *n* = 8) into the SNc. (E) PWT in NpHR. Light, F_(1, 16)_ = 22.33, *p* = 0.0002. (F) PWL in NpHR. Light, F_(1, 16)_ = 0.07, *p* = 0.79. (G) PWT in eYFP. Light, F_(1, 14)_ = 0.60, *p* = 0.45. (H) PWL in eYFP. Light, F_(1, 14) _= 0.003, *p* = 0.96. Two‐way ANOVA. (I,J) Representative image (I) and summary (J) showing that yellow light illumination increased c‐Fos(+) neurons in the STN. *t* = 6.74, *p* < 0.0001. Two‐tailed *t*‐test. (K,L) Representative traces and summary showing effects of yellow light illumination of the SNc^DA^‐STN projection on firing of STN neurons. F_(1.712, 17.12)_ = 9.54, *p* = 0.0023. *t* = 3.67, *p* = 0.013, light on versus baseline. *n* = 11. One‐way repeated measures ANOVA. (M,N) Schematic diagram, a representative image, and a summary for viral transfection of NpHR or eYFP into SNc DA neurons. (O–Q) A representative image and summary showing the effects of yellow light illumination of the right STN on mechanical PWT and thermal PWL on the contralateral side in NpHR (*n *= 8) and eYFP (*n* = 8) mice. (P) PWT. NpHR: *t* = 9.14, *P *< 0.0001; eYFP: *t* = 0.06, *p* = 0.95. (Q) PWL. NpHR: *t* = 0.21, *p* = 0.84; eYFP: *t* = 0.24, *p* = 0.81. Two‐tailed paired *t*‐test. (R–V) Schematic diagram, representative images, and summary showing chemogenetic inhibition of the SNc^DA^‐STN projection in hM4Di (*n* = 8) mice. (U) PWT. F_(3, 42)_ = 5.738, *p* = 0.002; *t* = 5.78, *p* = 0.004, baseline versus CNO; *t* = 8.17, *p* = 0.0005, CNO versus CNO + ropinirole; *t* = 0.69, *p* = 0.99, baseline versus CNO + ropinirole. (V) PWL: F_(3, 42)_ = 0.36, *p* = 0.78. Two‐way repeated measures ANOVA. * *P *< 0.05, ** *P *< 0.01, ns not significant.

Degeneration of SNc DA neurons is followed by complex modifications in the basal ganglion circuit [25, 26, 27, 28], which may be relevant to hyperalgesia in Parkinsonian pain [8, 23]. It has been mysterious whether transient dopamine deficiency without subsequent long‐term neuroplasticity in the basal ganglia circuit leads to hyperalgesia. To answer this question, we applied the optogenetic technique to inhibit SNc DA neurons and examined pain thresholds in mice. To achieve this goal, we injected AAV‐EF1α‐DIO‐NpHR‐eYFP or AAV‐EF1α‐DIO‐eYFP into the SNc of DAT‐Cre mice and implanted an optical fiber above the SNc (Figure 1A; Figure S1A). We confirmed that the viral vector transfected SNc DA neurons with high efficiency and specificity (Figure 1B,C), and in NpHR‐expressing SNc neurons, yellow light illumination (589 nm, 1 s, 1 mW) evoked an outward current in voltage‐clamp recording and inhibited firing in current‐clamp recording (Figure 1D). In pain behavioral examination, we observed that during yellow light illumination in the SNc, NpHR mice exhibited a lower mechanical paw withdrawal threshold (PWT) on the contralateral side, but not on the ipsilateral side (Figure 1E); interestingly, yellow light illumination of the SNc did not affect thermal paw withdrawal latency (PWL) on either side in NpHR mice (Figure 1F). In contrast, eYFP mice did not show alterations in either mechanical PWT (Figure 1G) or thermal PWL (Figure 1H) during yellow light illumination of the SNc. These pain behavioral outcomes may not be confounded by alteration in locomotion because yellow light illumination of the SNc did not change movement velocity in the open field test (Figure S1B). These data suggest that the basal activity of SNc DA neurons is important to maintain the mechanical threshold on the contralateral side.

Our previous studies have demonstrated that the hyperactivity in STN neurons is a common pathophysiology in Parkinsonian, inflammatory, and neuropathic pain [8, 23]. We wondered whether inhibition of SNc DA neurons enhances activity in STN neurons. Combining optogenetic inhibition and immunohistochemistry, we observed that repetitive inhibition of SNc DA neurons with the optogenetic technique (2 min 3 mW yellow light with 2 min intervals for 30 min) increased c‐Fos‐positive neurons in the STN (Figure 1I,J). These data suggest that inhibition of SNc DA neurons causes hyperactivity in STN neurons, while complex circuits, in addition to the SNc^DA^‐STN projection, may be engaged in an in vivo situation. We then performed patch‐clamp recordings from STN neurons in brain slices of DAT‐Cre mice 6 weeks after they received an injection of AAV‐EF1α‐DIO‐NpHR‐eYFP into the SNc. We found that yellow light illumination (5 s constant 2 mW) enhanced spontaneous firing in 73% (11 out of 15) of STN neurons among NpHR‐eYFP‐labeled fibers (Figure 1K,L). These data suggest that inhibition of the SNc^DA^‐STN projection is sufficient to enhance activity in STN neurons, and the STN may be a downstream nucleus mediating mechanical allodynia following inhibition of SNc DA neurons.

To confirm whether inhibition of the SNc^DA^‐STN projection is sufficient to induce mechanical allodynia, we injected AAV‐EF1α‐DIO‐NpHR‐eYFP or AAV‐EF1α‐DIO‐ eYFP into the SNc and implanted an optical fiber into the STN of DAT‐Cre mice (Figure 1M; Figure S1C). After confirming the specificity and efficiency of virus expression in SNc DA neurons (Figure 1N) and the presence of NpHR‐eYFP‐labeled fibers in the STN (Figure 1O), we applied optogenetic inhibition of the SNc^DA^‐STN projection by delivering yellow light into the STN and examined mechanical and thermal thresholds. We observed that yellow light illumination of the SNc^DA^‐STN projection led to a reduction in mechanical PWT on the contralateral hind paw in NpHR mice but not in eYFP mice (Figure 1P), no changes in thermal PWL on the contralateral hind paw in NpHR and eYFP mice (Figure 1Q), and no perturbation in locomotion in NpHR and eYFP mice (Figure S1D). These data confirm that the SNc^DA^‐STN projection mimics SNc DA neurons in pain modulation.

Considering that inhibition of SNc DA neurons may result in a reduction of dopamine level in their downstream circuits, to address which dopamine receptors mediate mechanical allodynia induced by SNc DA neuron inhibition, we intraperitoneally injected D1‐ or D2‐like receptor agonists and examined mechanical allodynia following inhibition of SNc DA neurons. We observed that mechanical allodynia on the contralateral hind paw following inhibition of SNc DA neurons was attenuated by intraperitoneal injection of ropinirole (a D2‐like receptor agonist) (1 mg/kg) (Figure S1E), but not by that of SKF38393 (a D1‐like receptor agonist) (10 mg/kg) (Figure S1F); mechanical threshold on the ipsilateral hind paw was not changed following inhibition of SNc DA neurons before and after intraperitoneal injection of either ropinirole (Figure S1G) or SKF38393 (Figure S1H). Thus, deactivation of D2‐like receptors may mediate mechanical allodynia on the contralateral side following inhibition of SNc DA neurons.

As in the above experiments, dopamine receptor agonists were administered systemically, they activate dopamine receptors in the STN and elsewhere. We then explored whether dopamine receptors in the STN mediate mechanical allodynia induced by inhibition of the SNc^DA^‐STN projection. We injected AAV‐EF1α‐DIO‐hM4Di‐mCherry into the SNc in DAT‐Cre mice and implanted a cannula above the STN (Figure 1R,S). This set of experiments allows stimulation of the SNc^DA^‐STN projection by injecting CNO into the STN via a cannula and activating dopamine receptors by injecting their agonists via the cannula (Figure 1T). Four weeks later, we injected CNO (3 µm, 200 nL) into the STN to inhibit the SNc^DA^‐STN projection and observed that the mice exhibited mechanical allodynia on the contralateral hind paw, but not on the ipsilateral hind paw; this effect was reversed by application of D2‐like dopamine receptor agonist (ropinirole, 200 nL, 1 µm) into the STN 15 min before (Figure 1U); we did not observed changes in thermal PWL on either hind paw before and after CNO or CNO+ropinirole (Figure 1V). These results suggest that inhibition of the SNc^DA^‐STN projection induces mechanical allodynia via deactivation of D2‐like receptors in the STN.

### 2.2 Stimulation of SNc DA Neurons and the SNc^DA^‐STN Projection Mitigates Mechanical Allodynia in Pain States

The proceeding data demonstrate the necessity of SNc DA neurons and the SNc^DA^‐STN projection to maintain normal pain threshold, we next wondered whether enhancing activity in SNc DA neurons and the SNc^DA^‐STN projection elevates pain thresholds. To address this issue, we subsequently applied the optogenetic technique to stimulate SNc DA neurons and the SNc^DA^‐STN projection and examined mechanical and thermal thresholds in physiological and pain states.

**FIGURE 2:**
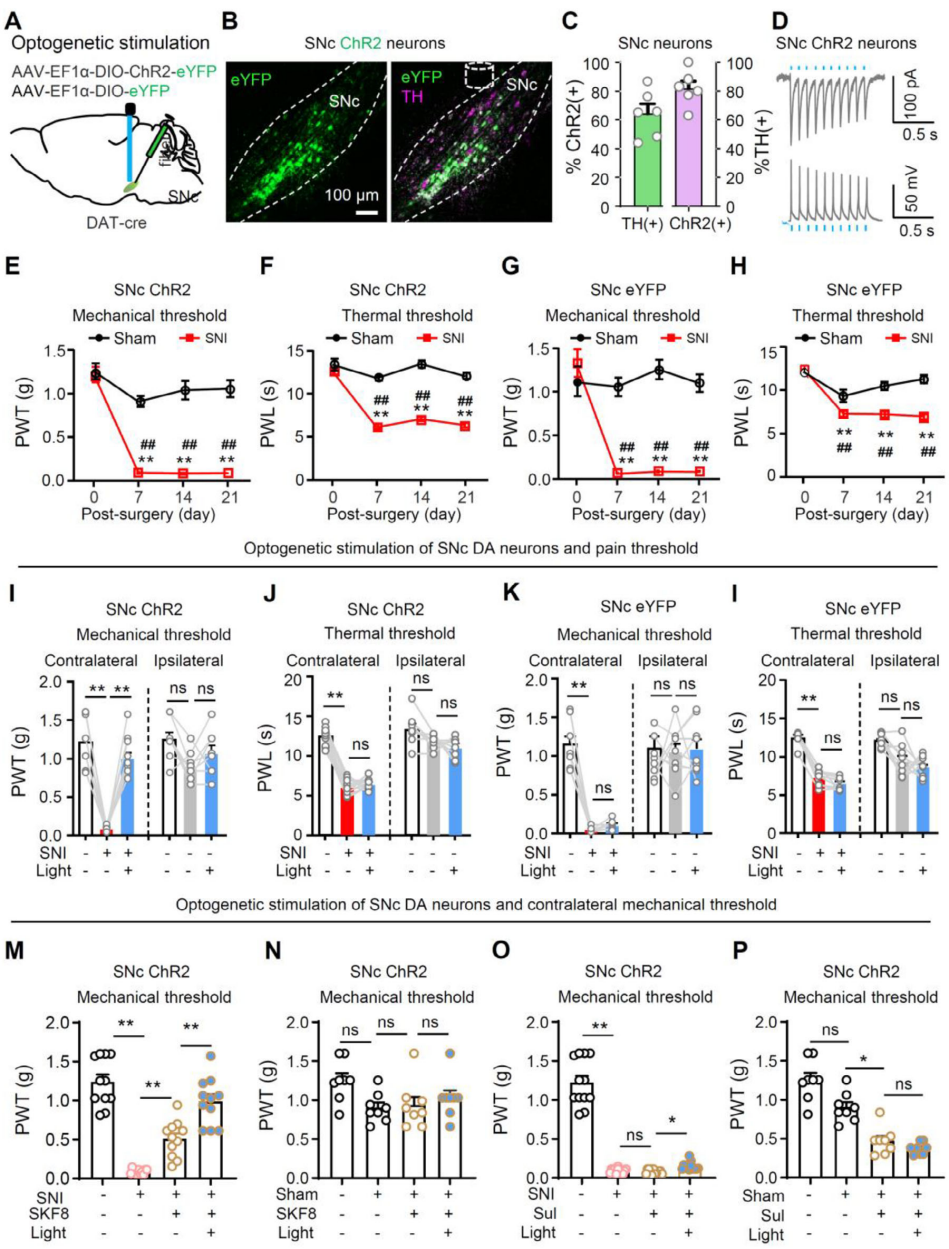
Stimulation of SNc DA neurons ameliorates mechanical allodynia in SNI mice through activation of D2 receptors. (A–D) Schematic diagram (A), representative images (B), summary of efficiency (green bar) and specificity (purple bar) of viral transfection (C), and typical traces (D) for optogenetic stimulation of ChR2‐expressing SNc DA neurons. (E–H) PWT and PWL on the left hind paw were measured in DAT‐Cre mice subjected to injection of AAV‐EF1α‐DIO‐ChR2‐eYFP (ChR2, *n* = 16)/AAV‐EF1α‐DIO‐eYFP (eYFP, *n* = 16) into the right SNc and spared nerve injury on the left sciatic nerve (SNI, *n* = 8) or sham operation (*n* = 8). (E) PWT in ChR2. Time: F_(1.77, 31.88)_ = 70.21, *P *< 0.0001; Group: F_(1, 18)_ = 148.7, *P *< 0.0001. (F) PWL in ChR2. Time: F_(2.434, 43.81)_ = 51.82, *p* < 0.0001; Group: F_(1, 18)_ = 301.2, *P *< 0.0001. (G) PWT in eYFP. Time: F_(3, 48)_ = 17.29, *p* < 0.0001; Group: F_(1, 16)_ = 116.5, *P *< 0.0001. (H) PWL in eYFP. Time: F_(2.844, 45.50)_ = 39.05, *p* < 0.0001; Group: F_(1, 16)_ = 42.58, *P *< 0.0001. (I–L) Effects of optogenetic stimulation of SNc DA neurons on mechanical PWT and thermal PWL on either hind paw in ChR2 (*n* = 8) and eYFP (*n* = 8) mice with or without blue light illumination of the right SNc. (I) ChR2 mice. Contralateral PWT: F_(1.416, 15.58)_ = 74.64, *p* < 0.0001. *t* = 12.63, *P *< 0.0001, SNI versus SNI+light. Ipsilateral PWT: F_(1.449, 10.15)_ = 3.60, *p* = 0.08. (J) ChR2 mice. Contralateral PWL: F_(1.635, 16.35)_ = 149.1, *P *< 0.0001. *t* = 0.89, *p* = 0.63, SNI versus SNI+light. Ipsilateral PWL: F_(1.400, 9.803)_ = 4.55, *p* = 0.05. *t *= 1.10, *p* = 0.52, SNI versus SNI+light. (K) eYFP mice. Contralateral PWT: F_(1.067, 8.535)_ = 112.8, *P *< 0.0001. *t* = 2.11, *p* = 0.13, SNI versus SNI+light. Ipsilateral PWT: F_(1.748, 13.99)_ = 0.043, *p* = 0.94. (L) eYFP mice. Contralateral PWL: F_(1.823, 14.58)_ = 183.6, *P *< 0.0001; *t *= 2.51, *p* = 0.07, SNI versus SNI+light. Ipsilateral PWL: F_(1.328, 10.62)_ = 10.78, *p* = 0.0051, *t* = 1.04, *p* = 0.77, SNI versus SNI+light. One‐way repeated measures ANOVA. (M–P) Effects of optogenetic stimulation of SNc DA neurons on mechanical PWT and thermal PWL on the contralateral hind paw in ChR2 mice (Control, sham or SNI) with or without blue light illumination of the right SNc before and after intraperitoneal injection of SKF83566 (SKF8, 0.03 mg/kg, *n *= 8) or sulpiride (Sul, 10 mg/kg, *n* = 12). (M) PWT in SNI mice. F_(2.046, 20.46) _= 50.99, *P *< 0.0001. *t* = 3.79, *p* = 0.02, SKF8 versus SKF8+light. (N) PWT in Sham mice. F_(2.609, 18.26)_ = 2.78, *p* = 0.08. (O) PWL in SNI mice. F_(1.080, 11.88)_ = 151.5, *P *< 0.0001. *t* = 3.85, *p* = 0.02, Sul versus Sul+light. (P) PWL in Sham mice. F_(2.289, 16.03)_ = 38.19, *P *< 0.0001, *t* = 1.35, *p* = 0.99, Sul versus Sul+light. * *P *< 0.05, ** *P *< 0.01, ns not significant. Two‐way repeated measures ANOVAs in (E–H). One‐way repeated measures ANOVAs in (I–P).

To fulfill optogenetic stimulation of SNc DA neurons, we injected AAV‐EF1α‐DIO‐ChR2‐eYFP or AAV‐EF1α‐DIO‐eYFP and implanted an optical fiber into the SNc in DAT‐Cre mice (Figure 2A). The viral vector transfected ChR2‐eYFP into SNc DA neurons with high efficiency and cell‐specificity (Figure 2B,C). Using patch‐clamp recording, we confirmed that blue light stimulation (2 ms, 1 mW, 10 Hz) evoked time‐locked inward currents in the voltage‐clamp mode and triggered spikes in the current‐clamp mode in ChR2‐eYFP‐labeled SNc DA neurons (Figure 2D). In pain behavior tests, we observed that in SNc‐ChR2 and SNc‐eYFP mice, blue light stimulation (5 ms, 20 Hz, 3 mW) of the right SNc did not change mechanical PWT (Figure S2A,D), thermal PWL (Figure S2B,E), and locomotion in the open field test (Figure S2C,F) in both ChR2 and eYFP mice. These data suggest that optogenetic stimulation of SNc DA neurons may not change pain thresholds in physiological conditions. We next examined pain thresholds in SNc‐ChR2 and SNc‐eYFP mice in acute and chronic pain states.

We subcutaneously injected capsaicin into the lower hind leg on either side to induce an acute inflammatory pain state which lasts for a few hours [24] and measured mechanical PWT 15 and 45 min after injection. Both SNc‐ChR2 (Figure S2G,I) and SNc‐eYFP (Figure S2H,J) mice received subcutaneous injection of capsaicin into the lower hind legs exhibited hypersensitivity to mechanical stimulation on hind paws. Blue light illumination of the SNc mitigated mechanical allodynia on the contralateral (left side) hind paw in SNc‐ChR2 mice (Figure S2G,I), but not in SNc‐eYFP mice (Figure S2H,J,), whereas it did not change mechanical allodynia on the ipsilateral (right side) hind paws in SNc‐ChR2 and SNc‐eYFP mice (Figure S2G–J). Additionally, optogenetic inhibition of SNc DA neurons did not alter mechanical allodynia on hind paws in the capsaicin‐induced inflammatory pain state in the same mouse cohort (SNc‐NpHR and SNc‐eYFP mice) as in Figure 1A–H and Figure S2K–N. These data indicate that stimulation of SNc DA neurons does not alter pain thresholds in physiological conditions, but mitigates mechanical allodynia on the contralateral side in an inflammatory pain state.

We then established a neuropathic pain mouse model with spared nerve injury of the sciatic nerve (SNI) on the left side and confirmed that both SNc‐ChR2 and SNc‐eYFP mice developed mechanical (Figure 2E,G) and thermal (Figure 2F,H) hypersensitivity on the hind paw of the injured side. We observed that blue light illumination of SNc DA neurons on the right side attenuated mechanical hypersensitivity on the hind paw of the SNI (left, contralateral) side, but not mechanical PWT on the intact (right, ipsilateral) side in SNc‐ChR2 mice (Figure 2I). We observed neither improvement in thermal hypersensitivity on the hind paw of the injured side (left, contralateral) nor changes in thermal PWL on the intact side (right, ipsilateral) in SNI mice upon optogenetic stimulation of SNc DA neurons on the right side (Figure 2J). Blue light illumination of the right SNc did not change mechanical (Figure 2K) and thermal (Figure 2L) thresholds on either hind paw in eYFP mice subjected to SNI surgery on the left side. Therefore, stimulation of SNc DA neurons also mitigates mechanical allodynia on the contralateral (injured) side in SNI mice.

We then performed pharmacological experiments to examine which dopamine receptors mediate the analgesic effect on the contralateral side by SNc DA neuron stimulation in SNI mice. We observed that in SNI mice, blocking D1‐like receptors with intraperitoneal administration of SKF83566 (SKF8, 0.03 mg/kg), a D1‐like receptor blocker, elevated mechanical PWT (Figure 2M), but did not block elevation of mechanical PWT by blue light stimulation of SNc DA neurons (Figure 2M); in sham mice, intraperitoneal administration of SKF83566 did not change mechanical PWT before and during blue light stimulation of SNc DA neurons (Figure 2N). On the contrary, blocking D2‐like receptors by intraperitoneally administering sulpiride (10 mg/kg, Sul), a D2‐like receptor antagonist, reduced mechanical PWT in sham mice (Figure 2P), but not in SNI mice (Figure 2O); it dramatically attenuated the effect of SNc DA neuron stimulation on mechanical PWT in SNI mice (Figure 2O), but did not change mechanical PWT in sham mice (Figure 2P). Note that after subjected to intraperitoneal injection of SKF8 and Sul, blue light illumination of the SNc did not change locomotion (Figure S2O,P). These data suggest that inhibition of D1‐ and D2‐like dopamine receptors affects mechanical PWT depending on pain states; SNc DA neurons exert an analgesic effect on the contralateral side in inflammatory and neuropathic pain mouse models via D2‐like dopamine receptors. However, the data do not clue where the contributing receptors locate.

**FIGURE 3:**
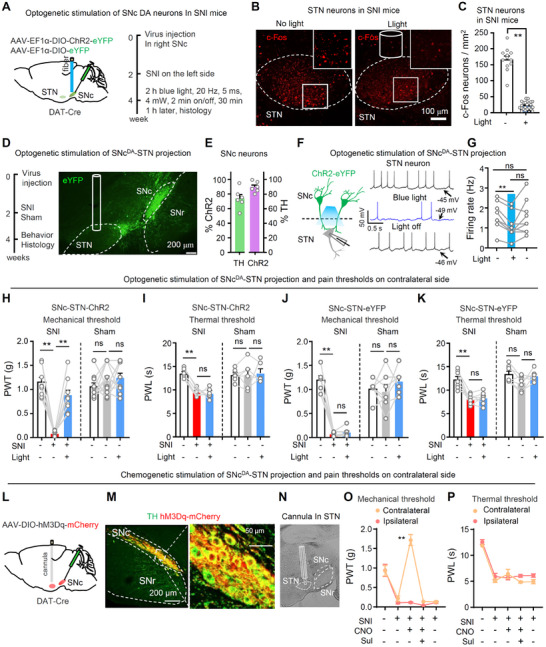
Stimulation of the SNc‐STN DA projection ameliorates mechanical allodynia in SNI mice through activating D2 receptors. (A–C) Schematic diagram, a representative image, and summary for optogenetic stimulation of SNc DA neurons and c‐Fos(+) neurons in the subthalamic nucleus (STN). *t* = 13.44, *p* < 0.0001. Two‐tailed unpaired *t*‐test. (D,E) Timeline, a representative image, and summary of efficiency (green bar) and specificity (purple bar) of viral transfection for optogenetic stimulation of the SNc^DA^‐STN projection. (F,G) Schematic diagram, representative traces, and summary showing the effect of blue light illumination of the SNc^DA^‐STN projection on firing in STN neurons. F_(1.179, 9.433)_ = 4.64, *p* = 0.05. *t* = 6.20, *p* = 0.0008, light on versus baseline. *n* = 9. One‐way repeated measures ANOVA. (H–K) Effects of blue light illumination of the right STN on PWT and PWL on the left hind paw of ChR2 (*n* = 9) and eYFP (*n* = 9) mice subjected to SNI or sham surgery. (H) ChR2 mice. PWT. SNI: *F* = 52.03, *P *< 0.0001; *t* = 10.83, *P* < 0.0001, control versus SNI; *t* = 7.60, *P* < 0.0001, SNI versus SNI light. Sham: *F* = 1.20, *p* = 0.32. (I) ChR2 mice. PWL. SNI: *F* = 26.65, *p* = 0.0025; *t* = 19.50, *P *< 0.0001, control versus SNI; *t* = 0.29, *p *= 0.95, SNI versus SNI light. Sham: *F* = 0.07, *p* = 0.89. (J) eYFP mice. PWT. SNI: *F* = 278.2, *P *< 0.0001; *t* = 16.10, *P *< 0.0001, control versus SNI; *t* = 1.64, *p* = 0.28, SNI versus SNI light. Sham: *F* = 1.13, *p* = 0.35. (K) eYFP mice. PWL. SNI: *F* = 17.74, *P *< 0.0001; *t *= 5.28, *p* = 0.0001, control versus SNI; *t* = 0.25, *p* = 0.96, SNI versus SNI light. Sham: *F* = 3.07, *p* = 0.09. One‐way repeated measures ANOVA. (L–N) Schematic diagram and representative images for chemogenetic stimulation of the SNc^DA^‐STN projection. (O,P) Effects of chemogenetic stimulation of the SNc^DA^‐STN projection on mechanical PWT and thermal PWL of either hind paw in bilateral SNI mice (*n* = 7). (O) PWT. Time: F_(1.749, 20.99)_ = 48.56, *P *< 0.0001. Contralateral: *t* = 5.67, *p* = 0.01, baseline versus CNO; *t* = 4.85, *p* = 0.03, SNI versus CNO; *t* = 2.21, *p* = 0.69, SNI versus CNO+sulpiride. Ipsilateral: *t* = 4.45, *p* = 0.04, baseline versus SNI; *t* = 3.40, *p* = 0.14, SNI versus SNI+CNO. (P) PWL. Time: F_(2.364, 28.37)_ = 83.09, *P *< 0.0001. Contralateral: *t* = 16.52, *P *< 0.0001, baseline versus SNI; *t* = 1.24, *p* = 0.99, SNI versus CNO. Ipsilateral: *t* = 11.35, *p* = 0.0003, baseline versus SNI; *t* = 0.44, *p* = 0.99, SNI versus SNI+CNO. Two‐way repeated measures ANOVAs. * *P *< 0.05, ** *P *< 0.01, ns not significant.

As STN neurons are hyperactive in SNI mice [23], and repetitive optogenetic stimulation (blue light, 5 ms, 20 Hz, 2 min on with 2 min intervals for 30 min) of SNc DA neurons transfected with ChR2‐eYFP (Figure 2A–D and Figure 3A) reduced the number of c‐Fos‐positive STN neurons in SNI mice (Figure 3B,C), we postulated that the SNc^DA^‐STN projection might mediate analgesic effects of SNc DA neurons. As c‐Fos‐staining lacks temporal resolution and is unable to exclude involvement of indirect circuit pathways in the modulation of STN neurons by SNc DA neurons, we applied the optogenetic technique to stimulate the SNc^DA^‐STN projection and examined the activity of STN neurons to confirm the contribution of the SNc^DA^‐STN projection to this process. We injected AAV‐EF1α‐DIO‐ChR2‐eYFP or AAV‐EF1α‐DIO‐eYFP into the SNc and implanted an optical fiber into the STN in DAT‐Cre mice (Figure 3D). After confirming the efficiency and specificity  transfection of ChR2‐eYFP into SNc DA neurons and presence of ChR2‐eYFP‐labeled fibers in the STN (Figure 3D,E), we recorded STN neurons in brain slices from ChR2 mice (Figure 3F) and observed that blue light illumination (2 ms, 2 mW, 20 Hz) (5 s) of the SNc^DA^‐STN projection, though did not evoke time‐locked postsynaptic currents in the STN, inhibited spontaneous firing in 60% (9/15) of STN neurons (Figure 3F,G). These data support that stimulation of the SNc‐STN projection is sufficient to inhibit STN neurons through an extra‐synaptic mechanism.

In behavioral tests, we observed that similar to optogenetic stimulation of SNc DA neurons, in naïve mice, stimulation of the SNc^DA^‐STN projection did not elevate mechanical PWT (Left panels in Figure S3A,B) and thermal PWL (Right panels in Figure S3A,B) on the contralateral side, and did not change locomotion (Figure S3C) in ChR2 and eYFP mice. Both ChR2 and eYFP mice exhibited a reduction in mechanical PWT and thermal PWL after SNI surgery, but not after sham surgery (Figure S3D–G). Blue light illumination of the SNc^DA^‐STN projection mitigated mechanical hypersensitivity on the contralateral side in ChR2 SNI mice (Figure 3H) but not in eYFP SNI mice (Figure 3J) without altering thermal hypersensitivity on the contralateral side in ChR2 (Figure 3I) and eYFP (Figure 3K) SNI mice. ChR2 and eYFP mice subjected to sham surgery did not show alterations in mechanical PWT and thermal PWL during blue light illumination of the SNc (Figure 3H–K). These results suggest that stimulation of the SNc^DA^‐STN projection exerts analgesic effects similar to that of SNc DA neurons.

Concerning whether D2‐like receptors also mediate the analgesic effect of the SNc^DA^‐STN projection, we performed two sets of experiments. First, in ChR2 SNI mice (Figure 3D), but not in ChR2 Sham mice, we observe that intraperitoneal administration of sulpiride (10 mg/kg) almost completely eliminated SNc^DA^‐STN projection stimulation‐induced mitigation of mechanical allodynia in SNI mice (Figure S3H,I). There exist at least two caveats in this set of experiments. On the one hand, it is possible that optogenetic stimulation of the SNc‐STN projection may evoke spikes on the axonal terminals which may backpropagate to the somata of SNc DA neurons and recruit downstream nuclei including the STN. On the other hand, systemically administered sulpiride blocks D2‐like receptors in the STN and elsewhere, and this strategy is not sufficient to address whether D2‐like receptors in the STN significantly contribute to the analgesic effect of stimulation of the SNc^DA^‐STN projection. To mitigate these caveats, we injected AAV‐EF1α‐DIO‐hM3Dq‐mCherry into the right SNc (Figure 3L,M) and implanted a cannula above the right STN in DAT‐Cre mice (Figure 3N). Four weeks after virus injection, stimulating the SNc^DA^‐STN projection by injecting 3 µm CNO (200 nL) into the right STN via the cannula increased mechanical PWT on the left hind paw in SNI (on the left side) mice; the analgesic effect of CNO was blocked by co‐application of 10 µm sulpiride into the STN (Figure 3O); injection of either CNO or CNO+sulpiride did not change thermal PWL in SNI mice (Figure 3P). These data suggest that D2‐like receptors in the STN play a significant role in mediating the analgesic effect of the SNc^DA^‐STN projection stimulation.

### 2.3 STN‐Projecting SNc DA Neurons Reduce Activity Upon Contralateral Mechanical Stimulation

**FIGURE 4:**
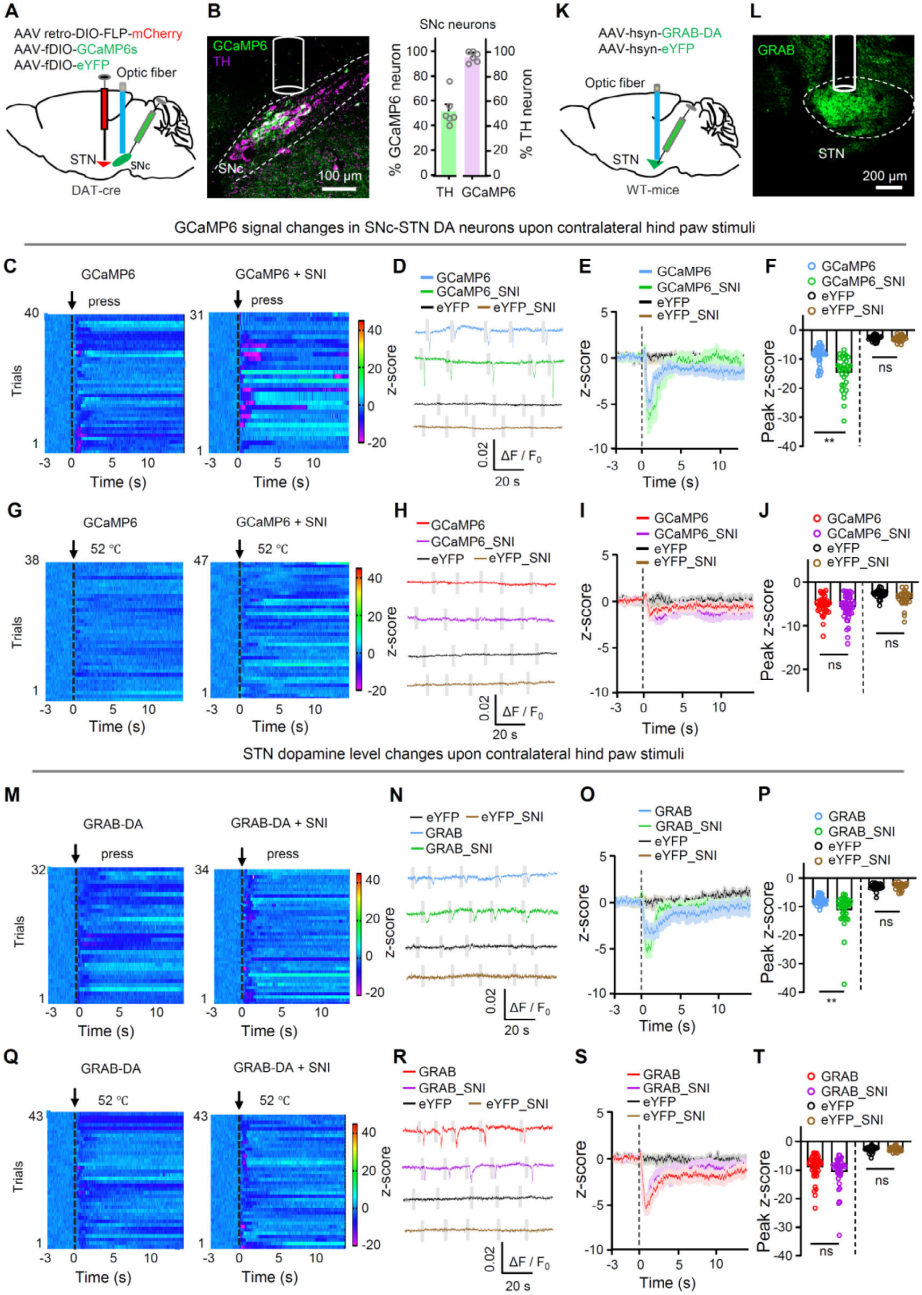
Mechanical pain stimulation decreases the activity of SNc‐STN DA neurons and dopamine release in the STN. (A,B) Schematic diagram (A) and a representative image (B, left panel) for fiber photometry recording of GCaMP6s signal in SNc‐STN DA neurons. (B, right panel) Summary of efficiency (green bar) and specificity (purple bar) of GCaMP6 transfection. (C–F) Heat maps, representative traces, averaged traces, and summary for contralateral mechanical stimulation‐induced responses in GCaMP6 and eYFP signal in the right SNc of sham and SNI mice. F_(3, 124)_ = 86.92, *P *< 0.0001. GCaMP6: *t* = 7.58, *P *< 0.0001, *n* = 40 in sham, *n* = 31 in SNI. eYFP: *t* = 0.22, *p* = 0.99, *n* = 40 in sham, *n* = 31 in SNI. (G–J) Heat maps, representative traces, averaged traces, and summary for contralateral thermal stimulation‐induced responses in GCaMP6 and eYFP signal in the right SNc of sham and SNI mice. F_(3, 132)_ = 12.86, *P *< 0.0001. GCaMP6: *t* = 0.93, *p* = 0.83, *n* = 38 in sham, *n* = 47 in SNI. eYFP: *t* = 1.88, *p* = 0.23, *n* = 38 in sham, *n* = 47 in SNI. (K,L) Schematic diagram and a representative image for fiber photometry recording of GRAB‐DA signal in the STN. (M–P) Heat maps, representative traces, averaged traces, and summary for contralateral mechanical stimulation‐induced responses in GRAB‐DA and eYFP signal in the STN of sham and SNI mice. F_(3,115)_ = 46.19, *P *< 0.0001. GRAB‐DA: *t* = 4.10, *p* = 0.0003, sham (*n* = 32) versus SNI (*n* = 34). eYFP: *t* = 0.25, *p* = 0.99, sham (*n* = 32) versus SNI (*n* = 34). (Q–T) Heat maps, representative traces, averaged traces, and summary for contralateral thermal stimulation‐induced responses in GRAB‐DA and eYFP signal in the STN of sham and SNI mice. F_(3,130)_ = 34.52, *P *< 0.0001. GRAB‐DA: *t* = 1.76, *p* = 0.29, sham (*n* = 41) versus SNI (*n* = 41). eYFP: *t* = 0.004, *P *> 0.999, sham (*n* = 43) versus SNI (*n* = 43). ** *P* < 0.01, ns not significant. 8 mice in each group, 3–5 trials were collected from each mouse. One‐way ANOVAs with Bonferroni tests for (F,J,P,T).

The above data demonstrate that the activity of SNc DA neurons and the SNc^DA^‐STN projection are involved in pain modulation. We wondered how STN‐projecting SNc DA (SNc‐STN DA) neurons responded to pain stimulation. To solve this puzzle, we applied fiber photometry to monitor intracellular calcium levels in SNc DA neurons (Figure 4A,B).

To perform fiber photometry recording from SNc‐STN DA neurons, we injected AAV retro‐hSyn‐DIO‐Flp into the right STN and injected AAV‐hSyn‐fDIO‐GCaMP6s (a genetically encoded green‐fluorescent calcium sensor) or AAV‐hSyn‐fDIO‐eYFP into the right SNc of DAT‐Cre mice and implanted an optical fiber into the right SNc (Figure 4A). In this set of experiments, about 50% of SNc DA neurons were transfected with GCaMP6 and about 95% of GCaMP6‐labeled SNc neurons are DA neurons (Figure 4B). As intracellular calcium accumulates when a neuron fires trains of action potentials, the activity of SNc‐STN DA neurons can be indirectly monitored by measuring GCaMP6 signal with fiber photometry [29, 30]. We applied mechanical pressure (200 g/mm^2^) onto hind paws of mice and observed that GCaMP6 signal in SNc DA neurons reduced dramatically upon pressure on the left (contralateral) hind paw (Figure 4C–F), but moderately upon pressure on the right (ipsilateral) hind paw (Figure S4A–D); it was not altered upon thermal stimulation on either contralateral (Figure 4G–J) or ipsilateral (Figure S4E–H) hind paw. We had two strategies to exclude the influence of motion artifacts on the pain response in the GCaMP6 signal. We corrected the GCaMP6 signal with isosbestic control at 405 nm before analyzed the data. Second, we measured GCaMP6 signal changes when the mice randomly lifted their hind paws during movement. We observed that the GCaMP6 signal in SNc DA neurons did not change when mice randomly lifted their paw (Figure S4Q). These results suggest that the mechanical stimulation‐induced reduction in GCaMP6 signal in SNc DA neurons may be related to pain sensation. Therefore, SNc‐STN DA neurons are involved in the processing of mechanical but not thermal pain preferentially on the contralateral side in physiological conditions.

We next examined whether these pain responses in the SNc‐STN DA neurons are enhanced in neuropathic pain. In SNI mice, mechanical pressure (on the injured side)‐induced reduction in GCaMP6 signal in contralateral SNc DA neurons was more dramatic than that in sham mice (Figure 4C–F). Thermal stimulation (on injured side)‐induced a minor reduction in GCaMP6 signal in contralateral SNc DA neurons, similarly in SNI and sham mice (Figure 4G–J). Additionally, mechanical and thermal stimulation on the hind paw on the surgery side in SNI and sham mice caused similar changes in GCaMP6 signal in ipsilateral SNc DA neurons (Figure S4A–H). These data suggest that SNI enhances pain response in SNc DA neurons to contralateral mechanical stimulation.

### 2.4 STN Dopamine Level Drops Upon Contralateral Mechanical Stimulation

**FIGURE 5:**
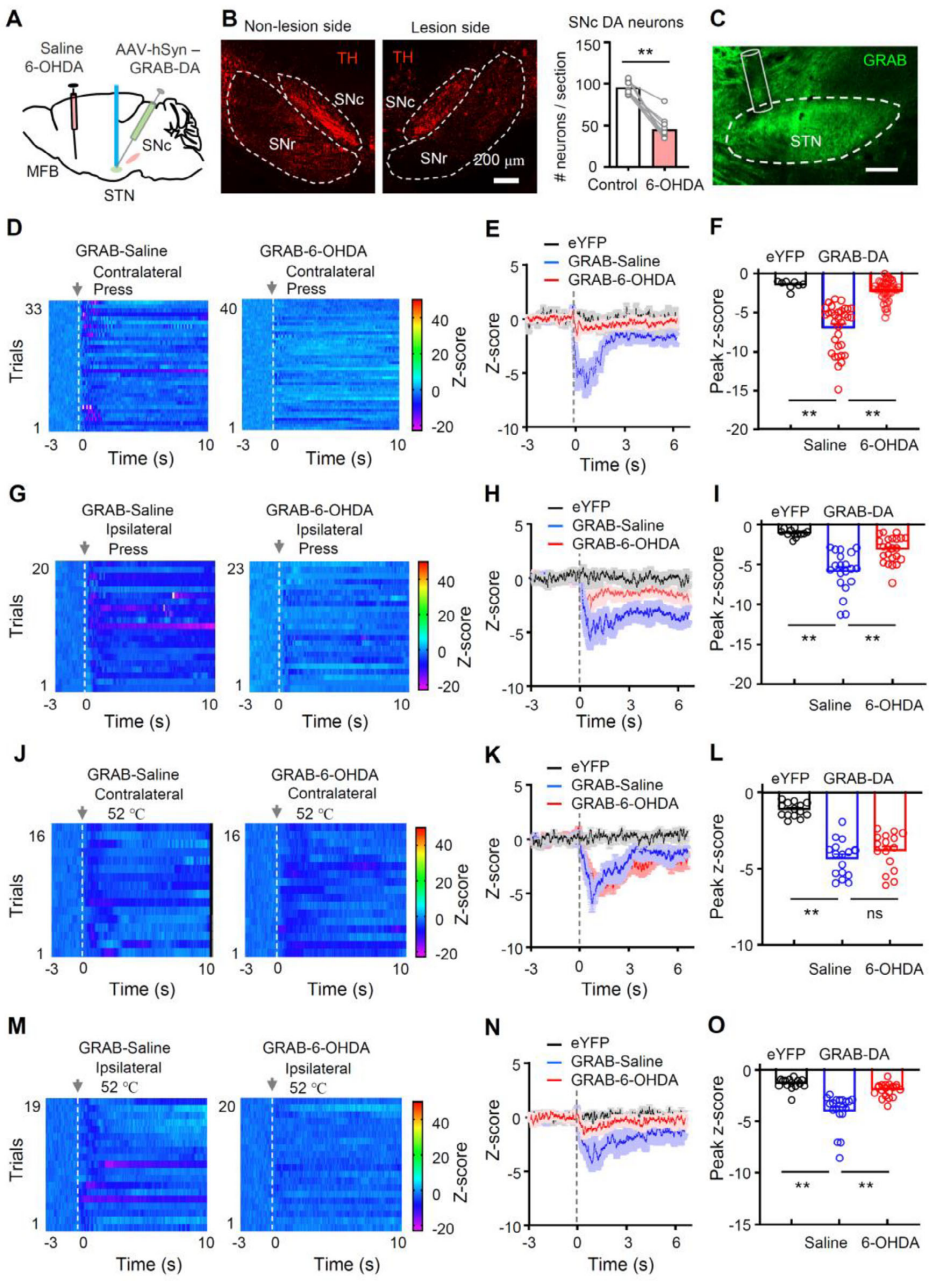
Alterations in dopamine level in the STN upon mechanical and thermal stimulation to bilateral hind paws in sham and hemiparkinsonian mice. (A) Mice were injected with AAV‐hSyn‐GRAB‐DA into the STN and saline or 6‐OHDA into the medial forebrain bundle (MFB) and were implanted with an optical fiber into the STN. (B) Representative images and summary showing damage of SNc DA neurons 2 weeks after 6‐OHDA injection. *t* = 9.61, *n* = 10, *P *< 0.0001, lesion versus non‐lesion side. Two‐tailed paired *t*‐test. (C) A representative image showing GRAB‐DA expression and a track of optical implant in the STN. (D–F) Heat maps, average traces, and a summary of peak z‐score showing reduction in STN dopamine level upon mechanical stimulation on the contralateral hind paw in GRAB‐DA mice subjected to either saline or 6‐OHDA injection. F_(2, 79)_ = 53.90, *P *< 0.0001. *t* = 9.72, *P *< 0.0001, 6‐OHDA (*n* = 33 trials) versus saline (*n* = 40 trials). (G–I) Heat maps, average traces, and a summary of peak z‐score showing reduction in STN dopamine level to mechanical stimulation onto the ipsilateral hind paw in eYFP and GRAB‐DA mice subjected to either saline or 6‐OHDA injection. F_(2, 52)_ = 25.78, *P *< 0.0001. *t* = 4.73, *p* < 0.0001, 6‐OHDA (*n* = 23 trials) versus saline (*n* = 20 trials). (J–L) Heat maps, average traces, and a summary of peak z‐score showing reduction in STN dopamine level to thermal stimulation onto the contralateral hind paw in eYFP and GRAB‐DA mice subjected to either saline or 6‐OHDA injection. F_(2, 43)_ = 40.13, *P *< 0.0001. *t* = 1.35, *p* = 0.46, 6‐OHDA (*n* = 16 trials) versus saline (*n* = 16 trials). (M–O) Heat maps, average traces, and a summary of peak z‐score showing reduction in STN dopamine level to thermal stimulation onto the ipsilateral hind paw in eYFP and GRAB‐DA mice subjected to either saline or 6‐OHDA injection. F_(2, 51)_ = 27.44, *P *< 0.0001. *t* = 5.71, *P *< 0.0001, 6‐OHDA (*n* = 20 trials) versus saline (*n* = 19 trials). ** *P *< 0.01. One‐way ANOVAs with Bonferroni tests for (F,I,L,O). 6 mice in each group. 3–7 trials were collected from each mouse.

As the SNc^DA^‐STN projection mimicked pain modulation by SNc DA neurons (Figures 1, 2, 3), it is likely that pain‐induced inhibition of SNc DA neurons may reduce dopamine level in the STN. To address this possibility, we next performed fiber photometry to monitor dopamine levels in the STN. To achieve this goal, we injected AAV‐hSyn‐GRAB‐DA (a genetically encoded green‐fluorescent dopamine sensor) [31, 32] or AAV‐CaMKII‐eYFP into the right STN of wild‐type mice and implanted an optical fiber at the injection site (Figure 4K,L). We observed that GRAB‐DA (dopamine) level in the right STN decreased upon mechanical (Figure 4M–P; Figure S4I–L) and thermal (Figure 4Q–T; Figure S4M–P) stimulation on contralateral (Figure 4M–T) and ipsilateral (Figure S4I–P) hind paws, but did not change upon random lift of either hind paw (Figure S4R). The reduction of dopamine level in the STN upon mechanical stimulation on contralateral, but not ipsilateral (, side was significantly enhanced in SNI mice (Figure 4M–P; Figure S4I–L); in contrast, the reduction of dopamine level in the STN upon thermal stimulation on contralateral (Figure 5Q–T) and ipsilateral (Figure S4M–P) hind paws was similar between SNI and sham mice. Therefore, decreases in activity of SNc‐DA neurons and dopamine level in the STN following mechanical pain stimulation on the contralateral hind paw are exaggerated in neuropathic pain. These results are consistent with the assumption that pain stimulation reduced the activity of SNc‐STN DA neurons and subsequently decreased dopamine levels in the STN. Note that upon pain‐like stimulation, changes of dopamine level in the STN were not identical to those of the activity of SNc DA neurons in terms of the modality and laterality of pain stimulation. These results may clue that pain stimulation‐induced reduction in STN dopamine level may involve the inhibition of the SNc^DA^‐STN projection and other neurotransmission inputs which may regulate dopamine level in the STN.

### 2.5 Activation of D2 Receptors in the STN Mitigated Mechanical Allodynia in Neuropathic Pain Mice

The above data suggest that decreased dopamine levels in the STN may result in hyperalgesia. Using fiber photometry recording, we observed that SNI mice showed reduced baseline GRAB‐DA signal in the STN than sham mice (Figure S5A–C), suggesting STN dopamine deficiency in SNI. We then implanted a cannula above the right STN to inject ropinirole (200 nL, 1 µm) or SKF38393 (200 nL, 10 µm) to increase the activation of D2‐ or D1‐like dopamine receptors (Figure S5D,E). In SNI mice, injection of ropinirole into the right STN elevated mechanical threshold on the left (injured, contralateral) but not right (ipsilateral) hind paw (Figure S5F), did not change thermal threshold on both hind paws (Figure S5G); injection of SKF38393 into the STN did not affect either mechanical (Figure S5H) or thermal (Figure S5I) threshold on both hind paws. In sham mice, injection of either ropinirole (Figure S5F,G) or SKF38393 (Figure S5H,I) into the STN did not change mechanical (Figure S5F,H) and thermal (Figure S5G,I) thresholds on both hind paws. These data suggest that reduction of dopamine levels followed by deactivation of D2‐like dopamine receptors may be an important pathophysiology for mechanical hypersensitivity in neuropathic pain.

### 2.6 Reduction in Dopamine Level and Deactivation of D2‐Like Receptors in the STN are Associated With Hyperalgesia in Parkinsonian Mice

To address whether SNc DA neurons are among the major sources of DA inputs to the STN, we injected retrograde virus (AAV retro‐hSyn‐DIO‐mCherry) into the STN of DAT‐Cre mice (Figure S6A). We observed mCherry‐labeled neurons in both the SNc and ventral tegmental area (VTA) (Figure S6B,C), and mCherry‐labeled SNc DA neurons outnumbered mCherry‐labeled VTA DA neurons (Figure S6D). Additionally, when we injected AAV‐EF1α‐DIO‐eYFP into the VTA of DAT‐Cre mice, we detected eYFP‐labeled fibers in the STN (Figure S6E). These data support that in addition to the SNc, the STN also receives DA inputs from the VTA. Consistent with previous studies, activity in VTA DA neurons is reduced upon pain stimulation [33, 34]. This may explain why the GRAB‐DA signal reduced upon both mechanical and thermal stimulation.

SNc DA neurons differ from VTA DA neurons not only in neuroanatomical profile, but also have a much higher susceptibility to neurodegeneration [35, 36]. Intracranial injection of 6‐OHDA into either the SNc, striatum, or medial forebrain bundle (MFB) has been widely used to establish Parkinsonian rodent models [1, 37]. Literature reports that in these models, SNc DA neurons are largely lesioned, leaving VTA DA neurons almost intact [8] (Figure S7A,B). Therefore, the Parkinsonian mouse model can be used to confirm whether SNc DA neurons provide major DA terminals in the STN responding to pain stimulation. For this purpose, we injected 6‐OHDA into the MFB to lesion the vulnerable SNc DA neurons by about 60%, relative to the non‐injection side, and injected AAV‐hSyn‐GRAB‐DA into the STN so that we could monitor dopamine level in the STN with fiber photometry (Figure 5A–C). Using a similar protocol, we previously observed that the GRAB signal in the STN was significantly reduced in 6‐OHDA mice [38]. In these mice, contralateral (Figure 5D–F) and ipsilateral (Figure 5G–I) mechanical stimulation and ipsilateral thermal stimulation (Figure 5M–O) induced less reduction in GRAB‐DA signal in the STN in 6‐OHDA mice than saline mice, but contralateral thermal stimulation induced a similar reduction in GRAB‐DA signal in the STN in 6‐OHDA and saline mice (Figure 5J–L). These data suggest that SNc DA neurons may significantly contribute to pain‐responding DA inputs to the STN, while thermal pain on the contralateral side may regulate distinct DA inputs from DA neurons resistant to 6‐OHDA lesion.

**FIGURE 6:**
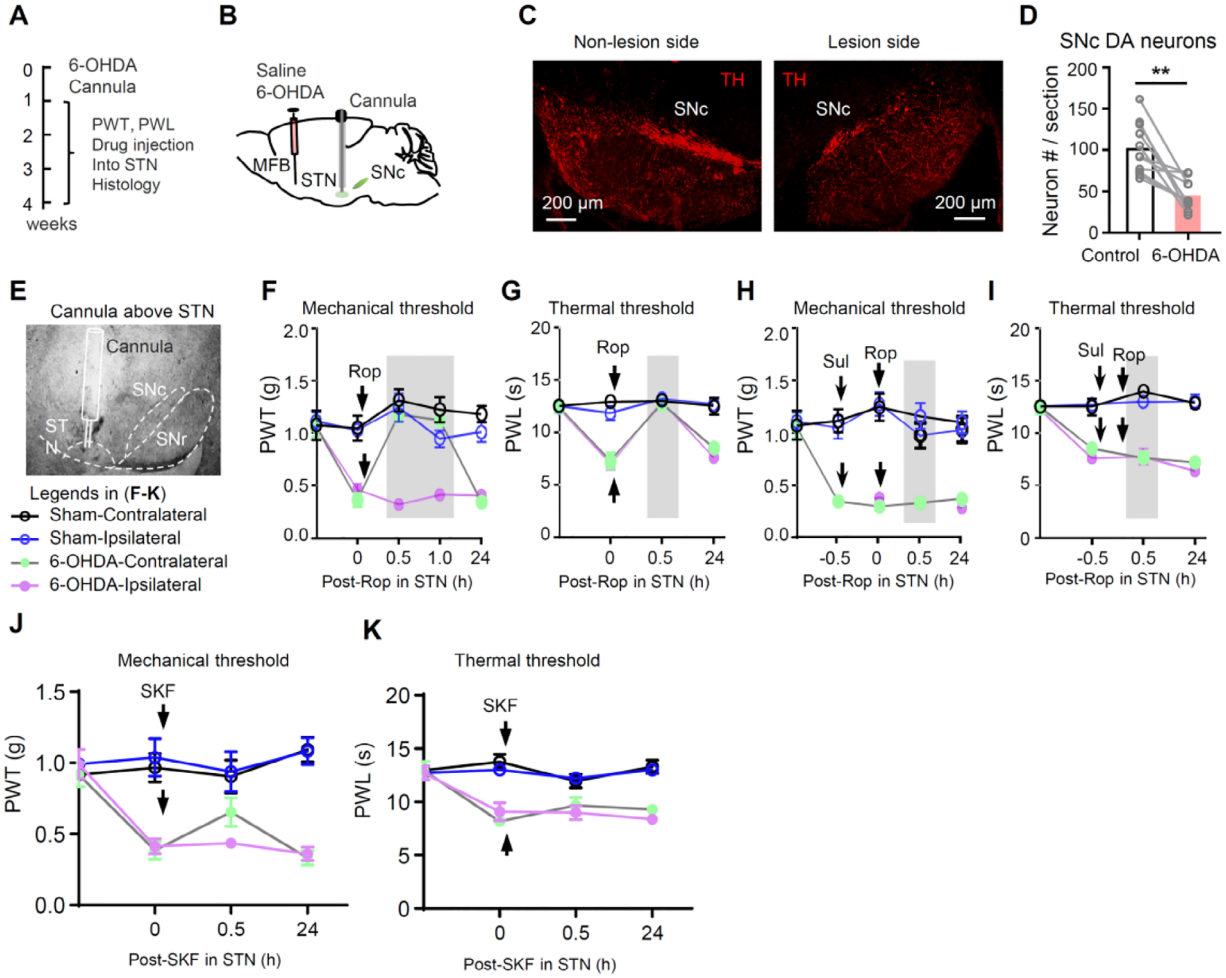
Activation of D2‐like receptors in the STN mitigates hyperalgesia in Parkinsonian mice. (A–D) Timeline (A), schematic diagram (B), representative images (C), and summary (D) for unilateral lesion of SNc DA neurons by 6‐OHDA injected into the medial forebrain bundle (MFB). *t* = 5.28, *P *< 0.0001, *n* = 11 from 5 mice. (E) A representative image showing the location of the cannula for drug administration into the STN. (F,G) PWT (F) and PWL (G) before and 2 weeks after 6‐OHDA or saline injection into the MFB, and after injection of ropinirole (Rop) into the STN. (F) PWT: Time, F_(3.206, 76.95)_ = 12.73, *P *< 0.0001; Group, F_(3, 24)_ = 77.33, *P *< 0.0001. (G) PWL: Time, F_(2.484, 59.62)_ = 39.34, *P *< 0.0001; Group, F_(3, 24)_ = 18.35, *P *< 0.0001. (H,I) PWT (H) and PWL (I) before and 2 weeks after 6‐OHDA or saline injection into the MFB, and after injection of sulpiride (Sul) and Rop into the STN. (H) PWT: Time, F_(2.489, 59.74) _= 13.33, *P *< 0.0001; Group, F_(3, 24)_ = 63.04, *P *< 0.0001. (I) PWL: Time, F_(2.929, 70.29)_ = 20.64, *P *< 0.0001; Group, F_(3, 24)_ = 68.69, *P *< 0.0001. (J,K) PWT (J) and PWL (K) before and 2 weeks after 6‐OHDA or saline injection into the MFB, and after injection of SKF38393 (SKF) into the STN. (J) PWT: Time, F_(3, 112)_ = 6.631, *p* = 0.0004; Group, F_(3, 112)_ = 29.13, *P *< 0.0001; *q* = 2.91, *p* = 0.17, contralateral 6‐OHDA. (K) PWL: Time, F_(3, 112)_ = 9.33, *P *< 0.0001; Group, F_(3, 112)_ = 28.38, *P *< 0.0001; *q* = 2.36, *p* = 0.35, contralateral 6‐OHDA. ** *P *< 0.01. 7 mice in each group. Two‐way repeated measures ANOVAs with Bonferroni tests for (F–K).

Our data demonstrate that SNc DA neurons, the SNc^DA^‐STN projection, and dopamine level in the STN are involved in pain modulation (Figures 1, 2, 3, 4; Figure S4) and suggest that reduction of dopamine level in the STN may be relevant to hyperalgesia in Parkinsonian pain. To address this issue, we implanted a cannula above the right STN in Parkinsonian mice established by injecting 6‐OHDA into the right MFB (Figure 6A–D). Consistent with our previous studies [8, 9, 38], these Parkinsonian mice developed persistent mechanical and thermal hyperalgesia on both hind paws, reflected by the reduction of bilateral mechanical PWT (Figure S7C,D) and thermal PWL (Figure S7E,F). After we injected ropinirole (0.3 µg, 200 nL) into the right STN (Figure 6E), we observed a significant increase in mechanical PWT on the contralateral (left) hind paw, but not on the ipsilateral (right) hind paw in 6‐OHDA mice (Figure 6F), and an elevation in thermal PWL on both hind paws in 6‐OHDA mice (Figure 6G). The injection of ropinirole into the right STN did not change either mechanical PWT (Figure 6F) or thermal PWL (Figure 6G) on either hind paw in sham mice. In 6‐OHDA mice, intraperitoneal injection of sulpiride (10 mg/kg) 30 min before ropinirole injection compromised the benefit of ropinirole injection into the right STN to mechanical hyperalgesia on the left side (Figure 6H) and thermal hyperalgesia on both sides (Figure 6I).

Different from the effect of ropinirole injection into the STN, injection of SKF38393 (10 µm, 200 nL) into the right STN had a marginal effect on mechanical PWT on the contralateral (left) hind paw in 6‐OHDA mice (Figure 6J), did not alter mechanical PWT on ipsilateral (right) hind paw (Figure 6J) and thermal PWL on either hind paw in 6‐OHDA mice (Figure 6K), and did not change mechanical PWT (Figure 6J) and thermal PWL (Figure 6K) on either hind paw in sham mice.

These results indicate that activation of D2‐like receptors in the STN is sufficient to alleviate mechanical hyperalgesia on the contralateral side and bilateral thermal hyperalgesia in Parkinsonian mice.

### 2.7 STN Neurons Receiving Projections From SNc DA Neurons Distinctly Regulate Contralateral Mechanical Threshold

**FIGURE 7:**
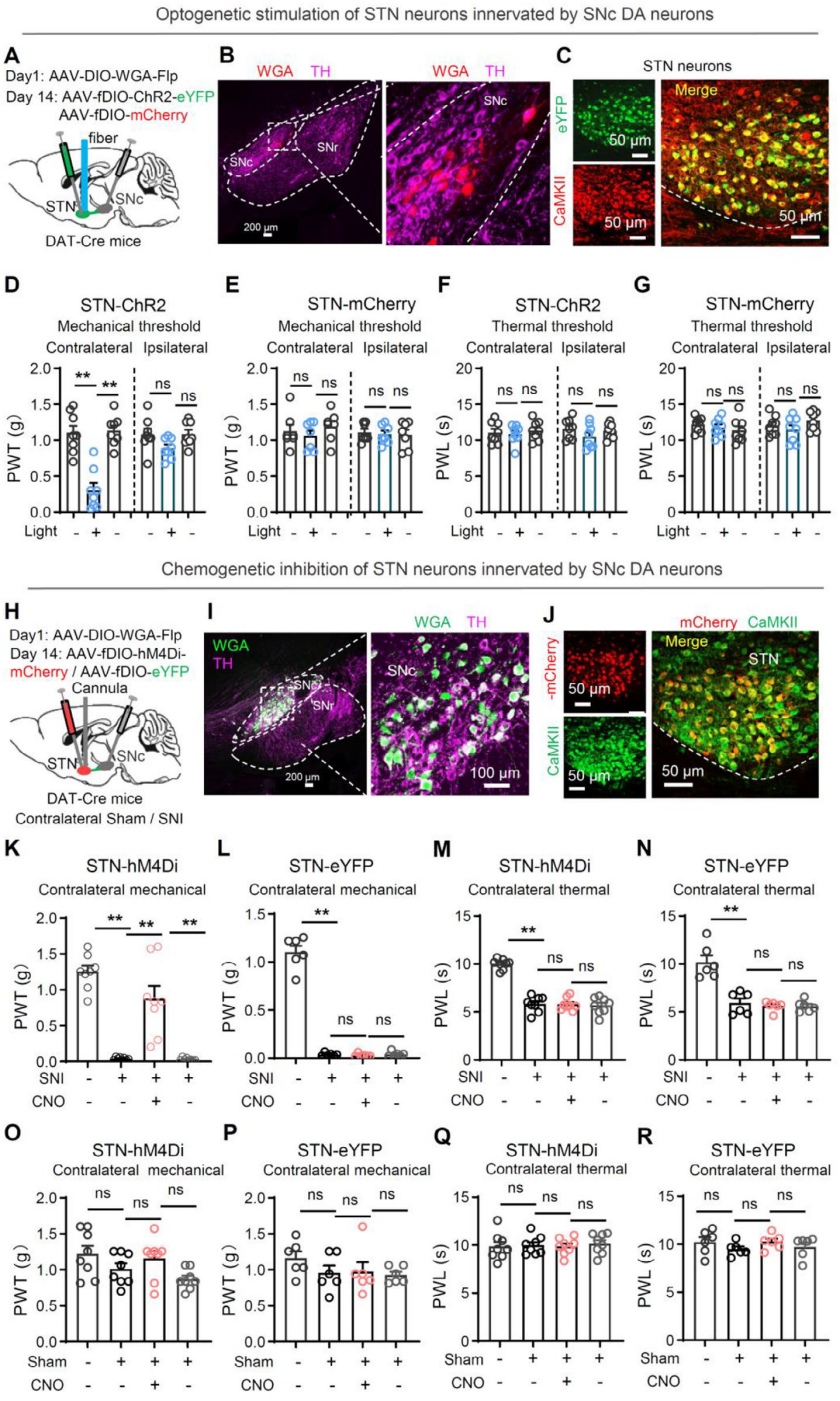
Bidirectional modulation of STN neurons innervated by SNc DA neurons regulates mechanical pain threshold on the contralateral side. (A–C) Schematic diagram and representative images for optogenetic stimulation of STN neurons receiving projections from SNc DA neurons. (D–G) Effects of blue light illumination of the STN on mechanical PWT and thermal PWL on either hind paw in ChR2 mice (*n* = 8) and mCherry mice (*n* = 7). (D) ChR2 mice. Contralateral PWT: F_(1.328, 9.295)_ = 41.07, *P *< 0.0001; *t* = 5.92, *p* = 0.002, control versus light. Ipsilateral PWT: F_(1.618, 11.33)_ = 3.34, *p* = 0.08. (E) mCherry mice. Contralateral PWT: F_(1.944, 11.66)_ = 0.76, *p* = 0.49; Ipsilateral PWT: F_(1.715, 10.29)_ = 0.07, *p* = 0.91. (F) ChR2 mice. Contralateral PWL: F_(1.804, 12.63)_ = 1.31, *p* = 0.30; Ipsilateral PWL: F_(1.680, 11.76)_ = 0.40, *p* = 0.64. (G) mCherry mice. Contralateral PWL: F_(1.756, 12.29)_ = 0.80, *p* = 0.46. Ipsilateral PWL: F_(1.091, 7.636)_ = 1.99, *p* = 0.20. (H–J) Schematic diagram and representative images for chemogenetic inhibition of STN neurons receiving projections from SNc DA neurons. hM4Di in the right STN and SNI or sham on the left side. (K–N) Mechanical PWT and thermal PWL on the left hind paw before and after CNO injection into the right STN in hM4Di mice (*n* = 8) and eYFP mice (*n* = 6) subjected to SNI surgery on the left side. (K) PWT in hM4Di mice. F_(1.327, 9.287)_ = 44.03, *P *< 0.0001; *t* = 15.13, *P *< 0.0001, baseline versus SNI; *t* = 4.71, *p* = 0.01, SNI versus CNO. (L) PWT in eYFP mice. F_(1.015, 5.076)_ = 212.4, *P *< 0.0001; *t* = 15.95, *p* = 0.0001, baseline versus SNI; *t* = 1.13, *p* = 0.99, SNI versus CNO. (M) PWL in hM4Di mice. F_(2.161, 15.13)_ = 102.6, *P *< 0.0001; *t* = 11.55, *P *< 0.0001, baseline versus SNI; *t* = 0.06, *p* = 0.99, SNI versus CNO. (N) PWL in eYFP mice. F_(1.271, 6.356)_ = 20.24, *p* = 0.003; *t* = 5.63, *p* = 0.01, baseline versus SNI; *t* = 0.70, *p* = 0.99, SNI versus CNO. (O–R) Mechanical PWT and thermal PWL in sham hM4Di mice (*n* = 8) and eYFP mice (*n* = 6) before and after CNO injection into the STN. (O) PWT in hM4Di mice. F_(2.203, 15.42)_ = 3.16, *p* = 0.07. (P) PWT in eYFP mice. F_(2.385, 11.92)_ = 1.03, *p* = 0.40. (Q) PWL in hM4Di mice. F_(1.893, 13.25)_ = 0.14, *p* = 0.86. (R) PWL in eYFP mice. F_(2.421, 12.10)_ = 0.91, *p* = 0.37. * *P *< 0.05, ** *P *< 0.01, ns not significant. One‐way repeated measures ANOVA with Bonferroni tests in (D–G, K–R).

There is a puzzle why stimulation of SNc DA neurons and the SNc^DA^‐STN projection exerts effects on mechanical and thermal pain thresholds in a pattern different from D2‐like receptor agonist injected into the STN. We propose that the difference may come from circuit components differentially affected by these modulations. To prove this, we used a transsynaptic viral vector to assist optogenetic modulation of STN neurons innervated by SNc DA neurons. To fulfill this goal, we injected a trans‐synaptic viral vector, AAV‐hSyn‐DIO‐WGA‐Flp, into the SNc in DAT‐Cre mice and AAV‐hSyn‐fDIO‐ChR2‐eYFP or AAV‐hSyn‐fDIO‐mCherry into the STN to label STN neurons receiving projections from SNc DA neurons, and implanted an optical fiber into the STN for optogenetic stimulation of these STN neurons (Figure 7A). After confirming expression of WGA in SNc DA neurons (Figure 7B) and ChR2‐eYFP in STN CaMKII neurons (Figure 7C), we performed optogenetic stimulation of STN neurons and examined mechanical and thermal thresholds. We observed that blue light illumination (5 ms, 4 mW, 20 Hz) of the STN significantly reduced mechanical PWT on the contralateral hind paw, but not on the ipsilateral hind paw in ChR2 mice (Figure 7D), and did not change mechanical PWT in mCherry mice (Figure 7E); blue light illumination of the STN did not change thermal PWL on both hind paws in ChR2 (Figure 7F) and mCherry (Figure 7G) mice. These data are consistent with pain modulation by inhibiting SNc DA neurons and the SNc^DA^‐STN projection (Figure 1).

We next examined whether inhibition of STN neurons innervated by SNc DA neurons sufficiently mitigates neuropathic pain like inhibition of the general STN neuron population, reported previously [23]. We injected trans‐synaptic viral vector, AAV‐hSyn‐DIO‐WGA‐Flp, into the right SNc in DAT‐Cre mice and AAV‐hSyn‐fDIO‐hM4Di‐mCherry or AAV‐hSyn‐f (DIO‐eYFP into the right STN to label STN neurons receiving projections from SNc DA neurons (Figure 7H). Three weeks later, we performed sham or SNI surgery on the left side in these mice (Figure 7H). We confirmed that WAG was specifically expressed in SNc DA neurons (Figure 7I) and hM4Di‐mCherry was efficiently expressed in STN CaMKII neurons (Figure 7J). In pain behavioral tests, we observed that SNI mice exhibited mechanical and thermal hyperalgesia on the contralateral (left) hind paw in hM4Di (Figure 7K, M) and eYFP (Figure 7L, N) mice; chemogenetic inhibition of STN neurons receiving projection from SNc DA neurons (in the right hemisphere) by injecting CNO (3 µm, 200 nL) into the STN significantly elevated mechanical PWT on the left hind paw in hM4Di (Figure 7K), but not in eYFP mice (Figure 7L); inhibition of these neurons did not affect thermal hyperalgesia in hM4Di (Figure 7M) and eYFP (Figure 7N) mice. Inhibition of STN neurons innervated by SNc DA neurons did not change mechanical (Figure 7O,P) and thermal pain thresholds (Figure 7Q,R) on the contralateral (left) hind paw in sham mice.

**FIGURE 8:**
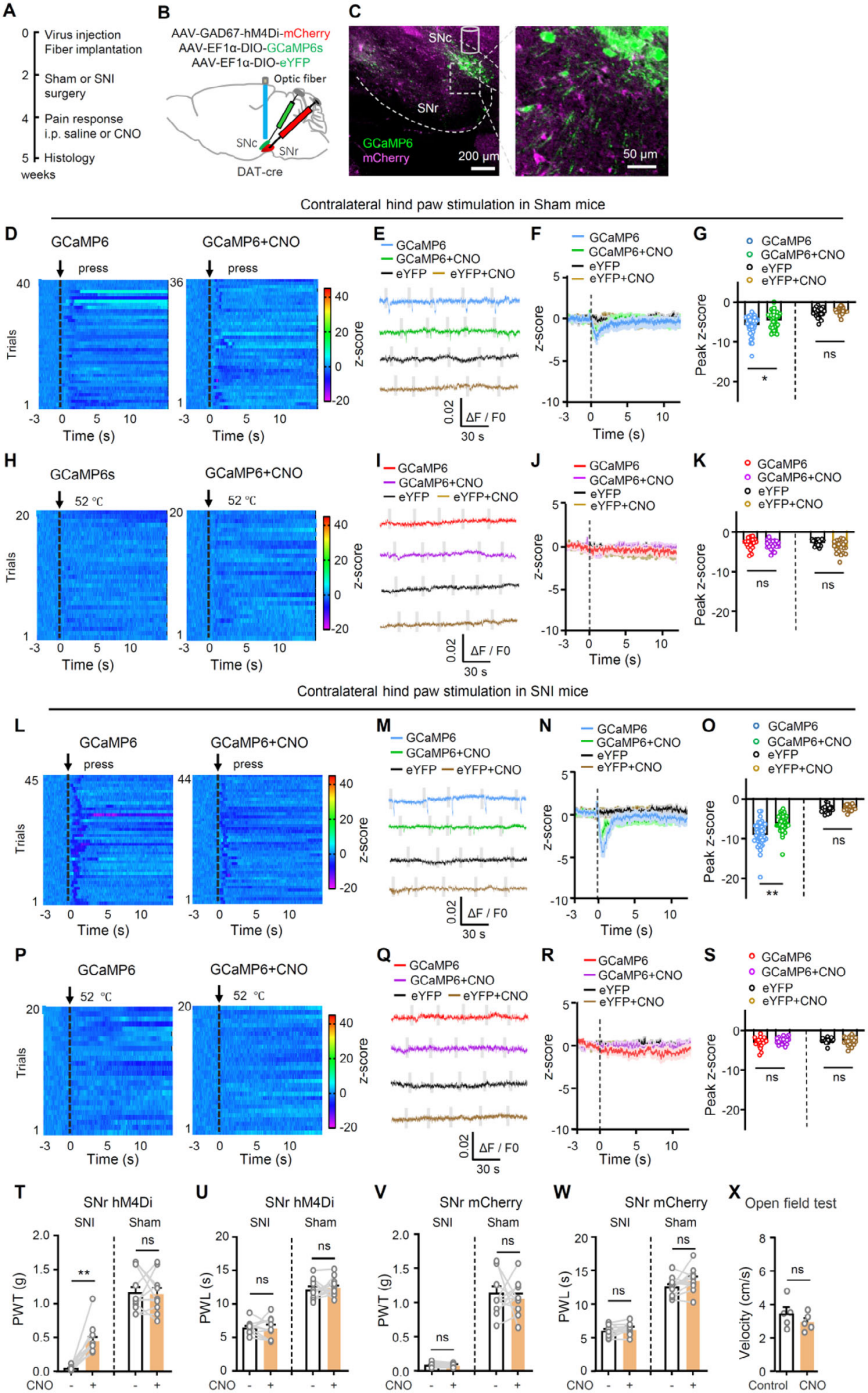
Inhibition of SNr GABAergic neurons attenuates pain response in SNc DA neurons. (A–C) Timeline, schematic diagram, and representative images for fiber photometry recording of GCaMP6s and eYFP signal in SNc DA neurons (green) and chemogenetic inhibition of SNr GABAergic neurons (magenta) in the right hemisphere. The mice subjected to mechanical and thermal stimulation and SNI/sham surgery on the left (contralateral) side. (D–G) Heat maps, representative traces, averaged traces, and a summary of peak responses in SNc GCaMP6 or eYFP signal to contralateral mechanical stimulation without or with inhibition (CNO) of SNr GABAergic neurons in sham mice. F_(3, 112)_ = 20.33, *P *< 0.0001. *t* = 2.72, *p* = 0.015, GCaMP6 versus GCaMP6+CNO (*n* = 40). eYFP: *t* = 0.89, *p* = 0.61, eYFP versus eYFP+CNO (*n* = 20). (H–K) Heat maps, representative traces, averaged traces, and a summary of peak responses in SNc GCaMP6 and eYFP signal to contralateral thermal stimulation without or with inhibition (CNO) of SNr GABAergic neurons in sham mice. F_(2.520, 47.88)_ = 2.79, *p* = 0.07. GCaMP6 versus GCaMP6_CNO (*n* = 20). (L–O) Heat maps, representative traces, averaged traces, and a summary of peak responses in SNc GCaMP6 and eYFP signal to contralateral mechanical stimulation without or with (CNO) inhibition of SNr GABAergic neurons in SNI mice. F_(3, 125)_ = 64.57, *P *< 0.0001. *t* = 6.13, *P *< 0.0001, GCaMP6 versus GCaMP6+CNO (*n* = 45); *t* = 0.26, *p* = 0.96, eYFP versus eYFP+CNO (*n* = 20). (P–S) Heat maps, representative traces, averaged traces, and a summary of peak responses in SNc GCaMP6 and eYFP signal to contralateral thermal stimulation without or with (CNO) inhibition of SNr GABAergic neurons in SNI mice. F_(2.379, 45.20)_ = 1.13, *p* = 0.34. GCaMP versus GCaMP6+CNO (*n* = 20). (T,U) PWT (T) and PWL (U) on the SNI and sham surgery side (left) in hM4Di mice 45 min after intraperitoneal injection of saline or CNO. (T) PWT. F_(2.453, 24.53)_ = 75.38, *P* < 0.0001. SNI: *t* = 5.76, *p* = 0.0004, *n* = 11; sham: *t* = 0.19, *p* = 0.98, *n* = 11. Two‐tailed paired *t‐*test. (U) PWL. F_(1.492, 14.92)_ = 84.76, *p* < 0.0001. SNI: *t* = 0.29, *p* = 0.95, *n* = 11; sham: *t* = 0.60, *p* = 0.81, *n* = 11. (V,W) PWT (V) and PWL (W) on the SNI and sham surgery side (left) in SNr mCherry mice 45 min after intraperitoneal injection of saline or CNO. (V) PWT. F_(2.228, 22.28)_ = 67.01, *P* < 0.0001. SNI: *t* = 0.05, *p* = 0.99, *n* = 11; sham: *t* = 0.60, *p *= 0.81, *n* = 11. (W) PWL. F_(1.955, 19.55)_ = 108.1, *p* < 0.0001. SNI: *t* = 0.86, *p* = 0.65, *n* = 11; sham: *t* = 1.65, *p* = 0.24, *n* = 11. (X) Velocity in the open field tests in hM4Di mice. *t* = 1.57, *p* = 0.10, n = 5. ** *P *< 0.01, ns not significant.

These data suggest that there is a group of STN neurons receiving projections from SNc DA neurons distinctly modulated contralateral mechanical pain threshold. This may be inconsistent with previous studies showing that STN neurons regulate mechanical and thermal thresholds on both hind paws [8, 23, 39]. However, STN neurons affect different pain modality and laterality through their divergent projections: regulating bilateral mechanical threshold through their projections to the globus pallidus interna and ventral pallidum, bilateral thermal threshold through their projections to the substantia nigra pars reticulata [8], while contralateral mechanical and thermal threshold via their projections to the lateral parabrachial nucleus [23]. We analyzed projections of STN neurons innervated by SNc DA neurons labeled as Figure 7H–J (Figure 8A) and observed that these neurons sent projections to the globus pallidus externa and interna, lateral and medial parabrachial nucleus, caudate putamen, and ventral pallidum (Figure S8, Supporting Information). We postulate that pain modulation effects by these STN neurons may result from the integrity of their divergent projections. Further investigations are needed to elucidate the neural circuit basis of this distinct pattern in which STN neurons innervated by SNc DA neurons modulate pain.

### 2.8 Pain Responses in SNc‐STN Neurons are Controlled by SNr GABA Neurons

Considering that SNc DA neurons responded to pain stimulation with a reduction in activity, they may receive a pain signal from upstream inhibitory neurons. To probe the upstream neurons of SNc‐STN DA neurons, we used a retrograde trans‐synaptic tracing strategy with rabies virus and its helper viral vectors [23, 40]. We injected AAV‐EF1α‐DIO‐TVA‐eGFP and AAV‐EF1α‐DIO‐oRVG into the SNc of DAT‐Cre mice and two weeks later, we injected RV‐CSV‐∆G‐tdTomato into the STN (Figure S9A). The mice were sacrificed in 10 days. We observed that a great majority of neurons (80.6% ± 3%, *n *= 5 slices from 5 mice) transfected with TVA‐eGFP were DA neurons identified with tyrosine hydroxylase (TH)‐antibody staining (Figure S9B–D, left panel); eGFP‐labeled neurons accounted for 44.4% ± 4.3% (*n* = 5 slices from 5 mice) of TH(+)‐DA neurons (Figure S9D, right panel); tdTomato(+) neurons were detected in the substantia nigra pars reticulata (SNr), but did not express TH (Figure S9E–G).

Given that the principal SNr neurons are GABAergic [41, 42], to confirm whether STN‐projecting SNc DA neurons are innervated by SNr neurons, as illustrated in Figure S9H, we injected AAV retro‐hSyn‐DIO‐mCherry into the STN in DAT‐Cre mice to label STN‐projecting SNc DA neurons with mCherry (Figure S9I,J) and injected AAV‐GAD67‐ChR2‐eYFP into the SNr to label GABAergic neurons with ChR2‐eYFP (Figure S9K). Six weeks later, we performed patch‐clamp recording from ChR2‐eYFP‐labeled SNr neurons and mCherry‐labeled SNc DA neurons (Figure S9L). We observed that blue light illumination (460 nm, 1 s, 2 mW) induced inward current and blue light pulses (2 ms, 20 Hz, 2 mW) evoked time‐locked spikes in ChR2‐eYFP‐labeled SNr neurons (Figure S9M); blue light illumination (460 nm, 20 Hz, 2 ms, 2 mW) of the SNr‐SNc projection evoked time‐locked outward currents in the voltage‐clamp mode (Figure S9N,O, upper panel) and inhibited firing rate in the current‐clamp mode (Figure S9N,P, lower panel) in mCherry‐labeled‐SNc‐STN DA neurons. Therefore, SNr GABAergic neurons may control the activity in SNc‐STN DA neurons.

To address whether SNr GABAergic neurons responded to peripheral pain stimulation, we monitored the activity of SNr GABAergic neurons with fiber photometry. We transfected SNr GABAergic neurons with GCaMP6s or eYFP by injecting AAV‐GAD67‐GCaMP6s or AAV‐hSyn‐eYFP into the SNr in wild‐type mice and implanted an optical fiber into the SNr (Figure S10A,B). We observed that the GCaMP6 signal in SNr GABAergic neurons was enhanced upon mechanical (Figure S10C–F,K–N) and thermal (Figure S10G–J,O–R) stimuli on either hind paw. These data are consistent with a previous study showing that both mechanical and thermal stimuli enhance activity in SNr neurons [43]. Thus, the modality and laterality of pain responses in SNr GABAergic neurons are different from those in SNc DA neurons. This discrepancy may be explained by an assumption that the GCaMP6 signal in Figure S10C–R might be collected from SNr GABAergic neurons not limited to those sending projections to SNc DA neurons.

To address whether activation of SNr GABAergic neurons by pain stimulation is related to pain stimulation‐induced inhibition of SNc DA neurons, we monitored the activity of SNc DA neurons with fiber photometry and inhibited SNr GABAergic neurons with a chemogenetic technique. In this set of experiments, we transfected GCaMP6 into SNc DA neurons by injecting AAV‐EF1α‐DIO‐GCaMP6s into the right SNc of DAT‐Cre mice, meanwhile, injecting AAV‐GAD67‐hM4Di‐mCherry into the right SNr to transfect hM4Di into SNr GABAergic neurons (Figure 8A–C). We observed that GCaMP6 signal in SNc DA neurons was reduced by mechanical stimulation on the contralateral (left) hind paw, and this reduction was attenuated by chemogenetic inhibition of SNr GABAergic neurons with intraperitoneal injection of CNO (1 mg/kg) (Figure 8D–G); thermal stimulation on the contralateral (left) hind paw induced a mild response in SNc DA neurons in mice before and after intraperitoneal injection of CNO (Figure 8H–K). Similar effects of CNO on mechanical (Figure 8L–O) and thermal (Figure 8P–S) responses in SNc DA neurons were also observed in SNI mice. In the pain behavioral test, we observed that chemogenetic inhibition of SNr GABAergic neurons (expressing hM4Di‐mCherry) elevated mechanical threshold on the injured (contralateral) hind paw in SNI mice without changing that in sham mice (Figure 8T); it did not change thermal threshold on either hind paw in SNI and sham mice (Figure 8U). CNO did not change either mechanical (Figure 8V) or thermal (Figure 8W) threshold in mice subjected to viral transfection of mCherry into SNr neurons and SNI or sham surgery on the contralateral side. Additionally, chemogenetic inhibition of SNr GABAergic neurons did not affect locomotion (Figure 8X). These data suggest that excitation of SNr GABAergic neurons may partially contribute to the inhibition of SNc DA neurons in response to mechanical stimulation.

Therefore, SNc DA neurons may receive a proportion of pain signal from SNr neurons, and inhibition of this pathway sufficiently mitigates pain responses in SNc DA neurons.

## 3 Discussion

Midbrain DA neurons primarily locate in the ventral tegmental area (VTA), SNc, and A11 area in the thalamus. The role of VTA DA neurons in pain modulation is well‐documented [44]. VTA DA neurons innervate both medium spiny neurons expressing D1‐ or D2‐like dopamine receptors in the nucleus accumbens, exerting nociceptive and analgesic effects, respectively [45]. Alterations in neuronal activity and circuit plasticity in the VTA are involved in the pathophysiology of chronic pain [33, 45, 46, 47, 48]. DA neurons in A11 area project to the caudal part of the spinal trigeminal nucleus and spinal cord dorsal horn, initiate/maintain hyperalgesia in neuropathic pain through activating D1‐like receptors [49], while alleviate neuropathic pain by activating D2‐like receptors [9, 49, 50, 51]. However, it remains unclear whether and how SNc DA neurons modulate pain.

Using cell‐specific strategies, we demonstrate that SNc DA neurons modulate pain through the SNc^DA^‐STN pathway. Inhibition of SNc DA neurons or the SNc^DA^‐STN projection and stimulation of STN neurons innervated by SNc DA neurons induced mechanical allodynia on the contralateral side in naïve mice, while stimulation of SNc DA neurons or the SNc^DA^‐STN projection and inhibition of STN neurons innervated by SNc DA neurons mitigated mechanical allodynia on the contralateral side in both acute and chronic pain states. The outcomes following modulation of SNc DA neurons and the SNc^DA^‐STN projection were mediated by alterations in D2‐ but not D1‐like dopamine receptor activity. Additionally, mechanical stimulation on the contralateral side induced a reduction in the activity of SNc DA neurons and dopamine level in the STN. These pain responses were enhanced in SNI mice. Similar to Parkinsonian mice [38], SNI mice exhibited a reduced dopamine level in the STN. Compensating dopamine deficiency in the STN by injecting a D2‐like receptor agonist alleviated mechanical allodynia in both neuropathic pain and Parkinsonian mice. This study advances understanding of the function of SNc DA neurons in pain modulation, reveals dopamine deficiency in the STN as an important pathophysiological basis for hyperalgesia in SNI and Parkinsonian mice, and provides D2‐like dopamine receptors in the SNc^DA^‐STN pathway as potential targets for the treatment of mechanical allodynia in inflammatory, neuropathic, and Parkinsonian pain.

Furthermore, combining optogenetics, retrograde tracing, DAT‐Cre mice, and patch‐clamp recording we demonstrate that STN‐projecting SNc DA neurons were innervated by SNr GABAergic neurons. It suggests a possible circuit, consisting of SNr GABAergic neurons → SNc DA neurons → STN CaMKII neurons. In this circuit, both mechanical and thermal pain stimuli enhanced activity in SNr GABAergic neurons, while only mechanical stimulation inhibited SNc DA neurons. Thus, SNc DA neurons show pain responses different from SNr GABAergic neurons in terms of the modality of pain stimuli. However, inhibition of SNr GABAergic neurons did significantly attenuate the response in SNc DA neurons to mechanical pain and elevate the mechanical threshold in SNI mice. It is likely that there may exist a subgroup of SNr GABAergic neurons projecting to the SNc only responding to mechanical stimulation. Further investigations with cell‐specific retrograde circuit labeling are needed to clarify this possibility.

We observed that dopamine release in the STN was significantly reduced in response to either mechanical or thermal stimulation on either hind paw, in contrast to the pain responses in SNc‐STN DA neurons. This suggests that SNc DA neurons may not be the only sources of DA inputs to the STN or that DA terminals in the STN may be subjected to modulation by other neurotransmitters altered by pain stimulation. In neuropathic pain and Parkinsonian mice, pain responses in the STN dopamine level were differentially altered. In SNI mice, the reduction of STN dopamine level following contralateral mechanical stimulation, but not ipsilateral mechanical stimulation and bilateral thermal stimulation, was enhanced. In Parkinsonian mice, the reduction of dopamine release in the STN by contralateral or ipsilateral mechanical stimulation, and ipsilateral thermal stimulation, but not contralateral thermal stimulation was significantly attenuated. These results suggest that SNc DA neurons susceptible to 6‐OHDA lesion are among the major sources of DA inputs to the STN responding to bilateral mechanical stimulation and ipsilateral thermal stimulation, while the contralateral thermal pain response may involve DA inputs from 6‐OHDA‐resistant DA neurons. There are studies showing that individual SNc DA neuron subtypes expressing Anxa1, Calb1, or Vglut2 differ in vulnerability and play different roles in encoding acceleration, deceleration, reward, and aversion [52, 53]. Similarly, our data suggest that SNc DA neurons may be heterogeneous in encoding pain. A cell‐specific study reveals that activities in somata and axonal terminals of the mesolimbic dopaminergic system correlate well upon reward cue, but differ upon reward expectation, which may be interfered by recruited non‐dopaminergic systems [53]. Our data showing that SNc‐STN DA neurons and STN dopamine level differ in pain responses suggest that the nondopaminergic system in the STN may be recruited during pain processing to modulate dopamine release. Further studies are warranted to address this possibility.

Emerging evidence shows that the hyperactivity in STN neurons is a common pathophysiology related to mechanical and thermal hyperalgesia on both sides in several pain states, including acute inflammatory pain, chronic inflammatory pain, neuropathic pain, and Parkinsonian pain [8, 23, 24]. In the present study, we reported a group of STN neurons which are innervated by SNc DA neurons and specifically regulate the mechanical threshold on the contralateral side. We observed that optogenetic inhibition of SNc DA neurons or the SNc^DA^‐STN projection increased c‐Fos‐positive neurons in the STN and caused mechanical allodynia in naïve mice. These data suggest that dopamine level is important in controlling the activity of STN neurons and then pain threshold. Indeed, optogenetic stimulation of either SNc DA neurons or the SNc^DA^‐STN projection reduced c‐Fos‐positive STN neurons and improved mechanical allodynia in SNI mice. Combining the optogenetic technique and brain slice patch‐clamp recording, we showed that stimulation and inhibition of the SNc‐STN DA projection, respectively, inhibited and excited STN neurons. Therefore, reduction of STN dopamine level may be a key pathophysiology underlying hyperactivity in STN neurons in neuropathic pain and Parkinsonian pain. This is supported by our data showing that injecting a D2‐like receptor agonist (may inhibit STN neurons [54]), but not a D1‐like receptor agonist, into the STN mitigated mechanical allodynia in both SNI and Parkinsonian mice.

It is noteworthy that optogenetic stimulation of SNc DA neurons and the SNc‐STN DA projection and injection of ropinirole into the STN did not change either mechanical or thermal threshold in naïve mice, but differentially mitigates mechanical or/and thermal thresholds in capsaincin‐induced pain, neuropathic, and/or Parkinsonian pain states. It suggests that the SNc^DA^‐STN pathway may be subjected to modifications in pain states. Consistent with this notion, we observed that in SNI mice, pain stimulation induced a stronger reduction in the activity of SNc DA neurons and dopamine level in the STN; in parkinsonian mice, pain stimulation‐induced reduction in dopamine level was largely attenuated because of dopamine depletion; whereas activation of D2‐like receptors in the STN mitigated mechanical allodynia in SNI mice, and mechanical and thermal hyperalgesia in parkinsonian mice. These pathophysiological phenomena may be associated with modifications in synaptic inputs and outputs of the STN. For instance, Parkinsonian mice exhibited reduction of cortical glutamatergic inputs and GABAergic inputs, enhancement of histaminergic inputs, attenuation of STN projection to the SNr, enhancement of STN projection to the globus pallidual interna and externa [25, 26, 27, 28, 55, 56], and upregulation of D1 and D3 dopamine receptors in the STN [38]. In SNI mice, STN neurons received enhanced glutamatergic inputs from the anterior cingulate cortex and weakened GABAergic inputs from the SNr [24, 39], while sent stronger glutamatergic outputs to the lateral parabrachial nucleus [23]. Further investigations are needed to address hyperalgesia‐related STN circuit plasticity in Parkinsonian and neuropathic pain states.

There exist several limitations in this study besides those we discussed in the last paragraphs. First, sex difference has been reported in the prevalence of PD and the severity of pain symptoms in PD [14, 57, 58]. In this study, we only performed experiments on male mice and are unable to address whether the SNr‐SNc‐STN DA pathway has a different role in pain modulation and representation between male and female mice. Second, 6‐OHDA lesions the nigrostriatal DA pathway within a very short time‐window. The time course of the development of pain and motor deficit may overlap in this model, which is inconsistent with clinical findings, showing pain appears earlier than motor deficits. Parkinsonian model with a slower progress may be better to address the involvement of the SNc‐STN DA pathway in pain processing. Third, a technical limitation exists in chemogenetic inhibition of SNr GABAergic neurons which could not restrict the inhibition onto SNr GABAergic neurons projecting to the SNc.

In summary, we reveal that SNc DA neurons and the SNc‐STN DA projection regulate mechanical pain threshold on the contralateral side via activating D2‐like receptors in both physiological and chronic pain conditions; pain signal may be relayed in sequence through the SNr‐SNc‐STN pathway and their collateral projections and converge onto STN dopaminergic terminals and regulate dopamine release; in parkinsonian and neuropathic pain conditions, activation of D2‐like receptors in the STN may be potential therapeutic strategy for the treatment of mechanical hyperalgesia.

## 4 Methods Section

### 4.1 Animals

The care and use of animals and the experimental protocols used in this study comply with the Regulations for the Administration of Affairs Concerning Experimental Animals (1988) in China and were approved by the Institutional Animal Care and Use Committee and the Office of Laboratory Animal Resources of Xuzhou Medical University. C57BL/6J background DAT‐Cre (Stock no. 006302) and Vgat‐IRES‐Cre (stock no. 028862) mice were purchased from the Jackson Laboratory. Heterozygous transgenic mice were crossed with wild‐type C57BL/6J mice for breeding in the animal facility of Xuzhou Medical University. The male transgenic and C57BL/6J wild type mice (>8 weeks old) used for experiments were group housed (no more than 5 per cage) on a 12‐hour light/dark cycle with ad libitum access to water and food. All behavioral experiments were performed in the light cycle. Efforts were made to minimize animal suffering and to reduce the number of animals used.

### 4.2 Viral Vectors

The viral vectors purchased from Brain VTA (Wuhan, China) and Braincase (Wuhan, China) include AAV‐EF1α‐DIO‐ChR2‐eYFP, AAV‐EF1α‐DIO‐NpHR‐eYFP, AAV‐EF1α‐DIO‐eYFP, AAV retro‐hSyn‐DIO‐Flp, AAV‐hSyn‐fDIO‐GCaMP6, AAV‐GAD67‐hM4Di‐mCherry, AAV‐GAD67‐GCaMP6s, AAV‐hSyn‐eYFP, AAV‐EF1α‐DIO‐TVA‐eGFP, AAV‐EF1α‐DIO‐oRVG, RV‐CVS‐∆G‐tdTomato, AAV1/2‐hSyn‐DIO‐Flp, AAV‐hSyn‐fDIO‐ChR2‐eYFP, AAV‐hSyn‐fDIO‐hM4Di‐mCherry, AAV1‐hSyn‐fDIO‐eYFP, AAV‐hSyn‐fDIO‐mCherry, AAV‐hSyn‐GRAB‐DA3.0, and AAV‐hSyn‐DIO‐WGA‐Flp. The titers of AAV (adenovirus‐associated virus, serotype 2/9), AAV (serotype 1/2), and RV (Rabies virus) are respectively 1 × 10^12^ to 5 × 10^12^, 1 × 10^13^ to 2 × 10^13^, and 3 × 10^8^ vg/mL.

### 4.3 Intracranial Injection of Viral Vectors and Drugs

Male mice were deeply anesthetized with isoflurane (3% for induction, 1.5% for maintenance) and stabilized in a stereotaxic apparatus (RWD Life Science Co., Ltd, Shenzhen, China) with a heating pad. The surgery was performed as described previously [8, 24, 59]. Briefly, after disinfection, an incision was made in the midline of the skin above the skull, the position of the skull was adjusted to level the bregma and the lamda. A hole above the injection site was drilled, and a 10 µL Hamilton syringe with a 30 G needle was mounted on a micro‐syringe pump (KD Scientific, Holliston, MA, USA) to pump in and out viral vector or a drug solution. After lowering the tip of injection needle to the SNc (AP, −3.1 mm; ML, 1.5 mm; DV, 4.3 mm) (200 nL), STN (AP, −1.77 mm; ML, 1.5 mm; DV, 4.8 mm) (150 nL), or SNr (AP, −3.1 mm; ML, 1.5 mm; DV, 4.6 mm) (300 nL), viral vector or drug solution was injected at a speed of 50 nL/min. The needle remained in place for 5–10 min before it was gradually lifted at a speed of 0.3 mm/20 s till out of the brain.

For pharmacological manipulation, a stainless‐steel guide cannula (26G, RWD Life Science, Shenzhen, China) was implanted 500 µm above the STN (AP, −1.77 mm; ML, 1.5 mm; DV, 4.3 mm) because the injection needle for drug delivery was 500 µm longer than the cannula. The mice were anesthetized with 1% isoflurane, and 200 nL drug solutions were injected into the STN with a microinjection pump (KD Scientific, Holliston, USA) at a rate of 50 nL/min. The needle was kept in place at least 5 min after termination of the injection before it was removed. Behavioral tests were performed 15–20 min after the mice recovered from anesthesia.

For postoperative pain relief, meloxicam (4 mg/kg) (Aladdin Biochemical Technology, Shanghai, China) was added into drinking water for 3 days. The mice subjected to virus injection were allowed to recover for at least 3 weeks before the behavioral tests and optogenetic manipulation and morphological assay. The expression of viral vectors and the position of the optical fibers or cannula implants in mice were confirmed histologically after the experiments.

### 4.4 Optogenetic Manipulation

For photostimulation, optical fibers [200 µm in diameter, NA 0.37 (Inper, Hangzhou, China)] were implanted into the SNc (AP, −3.1 mm; ML, 1.5 mm; DV, 4.3 mm) or the STN (AP, −1.77 mm; ML, 1.5 mm; DV, 4.8 mm), and 473‐nm blue light pulses (5 ms, 20 Hz, 4 mW) or 589 nm yellow light (constant, 3 mW) were delivered into the SNc or STN from laser generators (Newdoon, Hangzhou, China; Inper, Hangzhou, China). All optogenetic manipulations were performed unilaterally on the right hemisphere.

### 4.5 Fiber Photometry

Mice were handled by an experimenter and habituated for more than 3 days (10 min per day). After the mice were adjusted to the test environment for at least 1 h, they were gently held by the experimenter and either mechanical stimulation (200 g/mm^2^ pressure) or thermal stimulation (52°C heating block) was applied on hind paws. Fiber photometry instrument (ThinkerTech, Nanjing, China) [60, 61] was used to monitor GCaMP6 and eYFP signals in SNc DA neurons or SNr GABAerigc neurons or GRAB and eYFP signals in the STN. This instrument was equipped with a 470 nm LED light source (adjusted to 20–40 µW) to excite the above‐mentioned fluorophores and a 405 nm LED light source (adjusted to 20–40 µW) to excite isosbestic signals used to correct interference caused by photobleaching and motion artifacts. Trials (3–5 for each mouse) of mechanical and thermal stimulation were applied on hind paws while the above‐mentioned fluorescent signals were simultaneously recorded, digitized, and saved in excel format with a software provided by the manufacturer (ThinkerTech, Nanjing, China). The data recorded 3 s before and 10 s after the application of mechanical or thermal stimulation were imported into Clampfit 10.7 (Molecular Devices, San Jose, CA, USA) for analysis. We analyzed the fluorescent signal 3 s before stimulation and calculated the mean (*F*_0_) and its standard deviation (SD), then transformed the fluorescent signal (F) into z‐scores (*F*–*F*_0_)/SD. The peak amplitude and area under the curve (AUC) in each trace of fluorescent signal corresponding to pain‐like response in each trial were calculated.

### 4.6 *von Frey* Filament Test

The *von Frey* filaments were used to measure the mechanical threshold of both hind paws of mice acclimatized in a test compartment on a wide gauge wire mesh supported by an elevated platform as described previously [8, 24, 62]. The *von Frey* filaments range in force from 0.008 g to 4 g. The 50% paw withdrawal threshold (PWT) was calculated using Dixon's up–down method [63].

### 4.7 Thermal Nociception Threshold

Thermal paw withdrawal latencies (PWL) in both hind paws were recorded with a plantar anesthesia tester (Boerni, Tianjin, China) in mice acclimatized in a test compartment on a glass surface [8, 24]. A heating light source was positioned over the plantar surface of the hind paw, and the withdrawal latency was measured. A 20 s cut‐off time was used to prevent potential tissue damage to the surface of the hind paw.

### 4.8 Capsaicin‐Induced Secondary Mechanical Hyperalgesia

Capsaicin (0.01%, 20 µL in 10% DMSO/saline) was injected subcutaneously in the lower hind leg to induce secondary mechanical hyperalgesia in the plantar area of the hind paw [64].

### 4.9 Spared Nerve Injury

Spared nerve injury (SNI), a neuropathic pain model, was established according to a previous report [65]. Mice were deeply anesthetized with isoflurane, and two branches of the sciatic nerve (the common peroneal and tibial nerves) were tightly ligated with two nylon sutures separated 4 mm apart, and 2 mm sections in between were removed, but another branch of the sciatic nerve (the sural nerve) was kept intact. Sham control mice were subjected to skin incision and dissociation of the sciatic nerve without nerve ligation and nerve severing. Mechanical and thermal thresholds of sham and SNI mice were measured by applying the *von Frey* filament and Hargreaves tests on the area of the hind paw innervated by the sural nerve.

### 4.10 6‐OHDA‐Lesion of SNc DA Neurons

We established a hemiparkinsonian mouse model according to previous studies [8, 54, 66]. Mice were deeply anesthetized with isoflurane and stabilized on a stereotaxic frame (RWD Life Science, Shenzheng, China) for intracranial microinjection (KD Scientific, Holliston, USA). 6‐OHDA (12 µg per µL, 0.4 µL) was injected into the right medial forebrain bundle (MFB) (AP: +0.5 mm; ML: 1.2 mm; DV: 4.8 mm). Control mice were subjected to the injection of 0.4 µL saline in the right MFB. To protect noradrenergic neurons from being lesioned by 6‐OHDA, desipramine (20 mg per kg) was injected intraperitoneally 30 min before 6‐OHDA injection.

### 4.11 Locomotion

Each mouse was placed in the center of a cylinder (30 cm in diameter, 40 cm in height) and allowed to roam freely for 20 min. The locomotor activity of these mice was recorded and analyzed with the Ethovision XT 14.0 software.

### 4.12 Histology

Mice were sacrificed in a CO_2_ chamber and then subjected to cardiac perfusion with phosphate‐buffered saline (PBS), followed by 4% paraformaldehyde (PFA) in PBS. Mouse brains were removed and post‐fixed in 4% PFA overnight at 4°C. For immunostaining [8, 54], brain sections were incubated in a blocking buffer containing 5% donkey serum and 0.1% Triton X‐100 for 90 min at room temperature. Then the sections were incubated with primary antibody diluted in blocking buffer for 24 h at 4°C (rabbit anti‐c‐Fos IgG, 1:1000, Cell Signaling Technology; Chicken anti‐TH (tyrosine hydroxylase) IgG, 1: 500, Aves Lab; Mouse anti‐CaMKII IgG, 1:300, Cell signaling Technology). After washing three times (10 min each) in PBS, the sections were incubated with secondary antibodies (Cy3‐conjugated donkey anti‐rabbit IgG, 1:500; Alexa 488‐, Cy3‐, and Alexa 647‐conjugated donkey anti‐chicken IgG, 1:500; Alexa 488‐ and Cy3‐conjugated donkey anti‐mouse IgG, 1:500, Jackson ImmunoResearch) for 90 min at room temperature. The sections were washed three times (10 min each) in PBS, dried in the dark, and then cover‐slipped in mounting medium (Meilunbio, Dalian, China).

The sections were imaged with a confocal microscope (LSM 880, Zeiss), and the images were processed with ImageJ (NIH, Bethesda, MD).

### 4.13 Brain Slice Electrophysiology

Brain slice electrophysiological recording was conducted with minor modifications according to previously reported methods [23, 27]. Parasagittal slices (300 µm thick) containing the SNc, SNr, and STN were prepared using a vibratome (Leica VT‐1200S, Nussloch, Germany) in an ice‐cold modified sucrose‐based artificial cerebral spinal fluid (sACSF) saturated with 95% O_2_/5% CO_2_ (carbogen), containing (in mm) 85 NaCl, 75 sucrose, 2.5 KCl, 1.25 NaH_2_PO_4_, 4 MgCl_2_, 0.5 CaCl_2_, 24 NaHCO_3_, and 25 glucose. The brain slices were transferred into carbogenated sACSF at 32°C and allowed to recover for 60 min, and then placed in normal carbogenated ACSF containing (mm) 125 NaCl, 2.5 KCl, 1.2 NaH_2_PO_4_, 1.2 MgCl_2_, 2.4 CaCl_2_, 26 NaHCO_3_, and 11 glucose at 26°C for at least 30 min prior to use.

Neurons in brain slices were visualized under an upright microscope (FN‐1, Nikon, Tokyo, Japan), equipped with a CCD‐camera (Flash 4.0 LTE, Hamamatsu, Hamamatsu city, Japan). Whole‐cell patch‐clamp recordings were obtained using a patch‐clamp setup composed of a dual‐channel MultiClamp 700B amplifier, a Digidata 1550B analog‐to‐digital converter, and pClamp 10.7 software (Molecular Devices, San Jose, CA, USA). The patch electrodes had a resistance of 4–6 MΩ when filled with a low‐chloride intrapipette solution containing (in mm) 135 K gluconate, 0.2 EGTA, 0.5 CaCl_2_, 10 HEPES, 2 Mg‐ATP, and 0.1 GTP, pH: 7.2; osmolarity: 290–300 mOsm. All recordings were performed at 32°C ± 1°C maintained by a dual‐channel temperature controller (TC‐344C, Warner Instruments, Holliston, MA, USA).

For light‐evoked responses, blue light (460 nm, 2 mW) was delivered through an optical fiber (200 µm, NA 0.37) connected to a PlexBright LED light source (Plexon Inc., Hong Kong, China). Light‐evoked inhibitory postsynaptic currents (eIPSCs) were recorded at −45 mV. To confirm whether GABAergic connections were involved, bicuculline (10 µm) was bath‐applied.

### 4.14 Chemicals

(E)‐Capsaicin, Clozapine‐N‐oxide (CNO), CNQX disodium, ropinirole, SKF38393, SKF83566, and sulpiride were purchased from MedChemExpress (Monmouth Junction, NJ, USA). 6‐hydroxyl dopamine hydrobromide (6‐OHDA), desipramine hydrochloride, and meloxicam were purchased from Aladdin Scientific (Shanghai, China).

### 4.15 Statistical Analysis

For optogenetic, fiber photometry, pharmacological, and behavioral experiments, mice were randomly assigned to experimental groups. For pain behavior assessments, the experimenters were blind to the chemicals and the viral vectors injected into the mice. GraphPad Prism (version 8.4) was used for all statistical analyses. All summarized data were expressed as mean ± SEM and scatter points in histograms. Two‐tailed paired or unpaired *t*‐tests, one‐way ANOVAs, one‐way repeated ANOVAs, two‐way ANOVA, two‐way repeated measures ANOVAs followed by post‐hoc pair‐wise comparisons were used to compare behavioral data, as indicated in the figure legends. If the data did not pass normal distribution and equal‐variance tests, statistical significance of the data was evaluated with the Mann–Whitney test or one–way or two‐way ANOVA rank tests. In all tests, a value of *p* < 0.05 was considered statistically significant.

## Author Contributions

C.X., C.Z., and G.C. designed the experiments. C.X., C.Z., G.C., and S.L. supervised this study. X.Y.X. and C.X. collected and analyzed the electrophysiological data. Y.J., S.Y.L., J.Q.Z., X.Y.X., and Y.Y.J. performed mouse survival surgeries and morphological experiments. Y.J., S.Y.L., J.Q.Z., C.Y., D.Y.L., M.N., Y.Y.J., and Y.N. performed the behavioral tests and illustrated the data. S.Y.L., Y.Y.J., and C.Y. managed the mouse colony. C.X., C.Z., J.Y., G.C., S.L., and B.Z. wrote and revised the manuscript. All authors read and approved the manuscript.

## Conflicts of Interest

The authors declare no conflict of interest.

## Supporting information




**Supporting File**: advs75182‐sup‐0001‐SuppMat.pdf.

## Data Availability

The data that support the findings of this study are available in the supplementary material of this article.
