# Supplementary material for: Nigra‐Subthalamic Dopaminergic Circuitry Modulates and Represents Distinct Pain Modality in Physiological and Pain States in Mice
Source: Adv Sci (Weinh). 2026 Apr 7:e19913. Online ahead of print. doi: 10.1002/advs.202519913 (PMC13334656; doi:10.1002/advs.202519913)
Supplement: Supplementary file 1 — Supporting File: advs75182‐sup‐0001‐SuppMat.pdf. [file ADVS-9999-e19913-s001.pdf]

Supplementary information

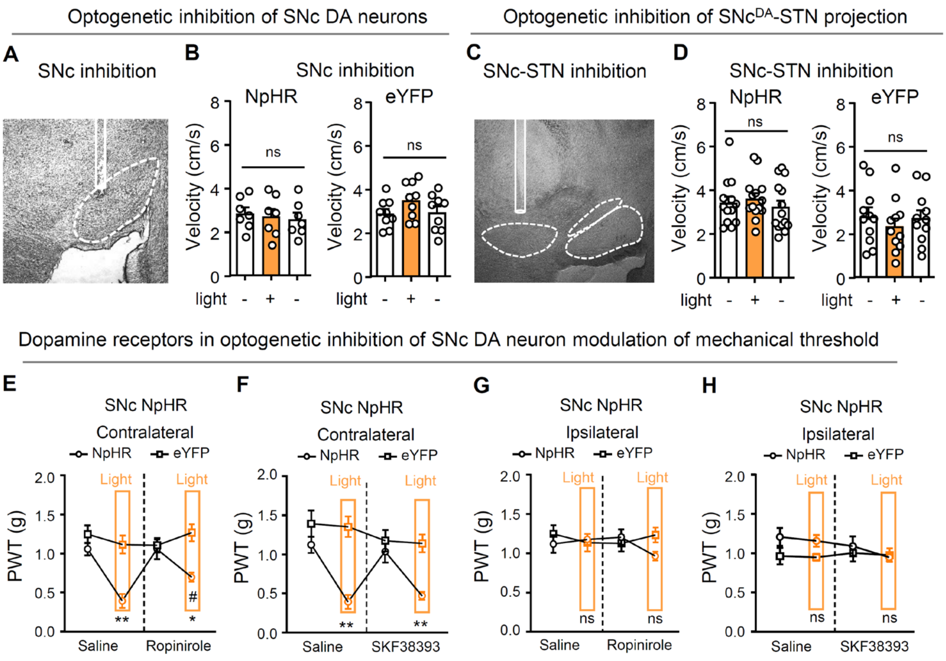

Figure S1 Optogenetic inhibition of either SNc DA neurons or the SNc-STN DA projection does not regulate locomotion in mice.

(A) A representative image showing the location of an optical fiber in the SNc for optogenetic inhibition of SNc DA neurons.

(B) Effects of yellow light illumination (yellow bar) of the SNc on velocity of locomotion in an open field arena in NpHR (*n* = 9) and eYFP (*n* = 7) mice. NpHR: $F_{(1.393, 11.15)} = 3.73$, $p = 0.07$. eYFP: $F_{(1.652, 9.912)} = 1.58$, $p = 0.25$. One-way repeated measures ANOVAs.

(C) A representative image showing the location of an optical fiber in the STN for optogenetic inhibition of the SNc-STN projection.

(D) Effects of yellow light illumination (yellow bar) of the STN on velocity of locomotion in an open field arena in NpHR (*n* = 14) and eYFP (*n* = 11) mice. NpHR: $F_{(1.681, 21.86)} = 1.10$, $p = 0.34$. eYFP: $F_{(1.306, 13.06)} = 2.41$, $p = 0.14$. One-way repeated measures ANOVAs

(E-H) Effects of yellow light illumination of the SNc on mechanical PWT and thermal PWL on either side in NpHR (*n* = 8) and eYFP (*n* = 8) mice before and after intraperitoneal injection of ropinirole and SKF38393. **(E)** Contralateral PWT and ropinirole. Time: $F_{(2.474, 34.64)} = 7.07$, $p = 0.0014$. Group: $F_{(1, 14)} = 19.47$, $p = 0.0006$. **(F)**

Contralateral PWT and SKF38393. Time: $F_{(2.380,\ 33.32)} = 7.07$, $p = 0.002$. Group: $F_{(1,\ 14)} = 23.58$, $p = 0.0003$. **(G)** Ipsilateral PWT and ropinirole. Time: $F_{(2.166,\ 30.33)} = 0.79$, $p = 0.79$. Group: $F_{(1,\ 14)} = 3.21$, $p = 0.10$. **(H)** Ipsilateral PWT and SKF38393. Time: $F_{(2.563,\ 35.88)} = 0.31$, $p = 0.79$. Group: $F_{(1,\ 14)} = 0.82$, $p = 0.38$.

* $p < 0.05$, ** $p < 0.01$, ns not significant, light vs no light; # $P < 0.05$ light in saline vs light in ropinirole. One-way repeated measures ANOVA for (**B, D**). Two-way repeated measures ANOVA for (**E-H**).

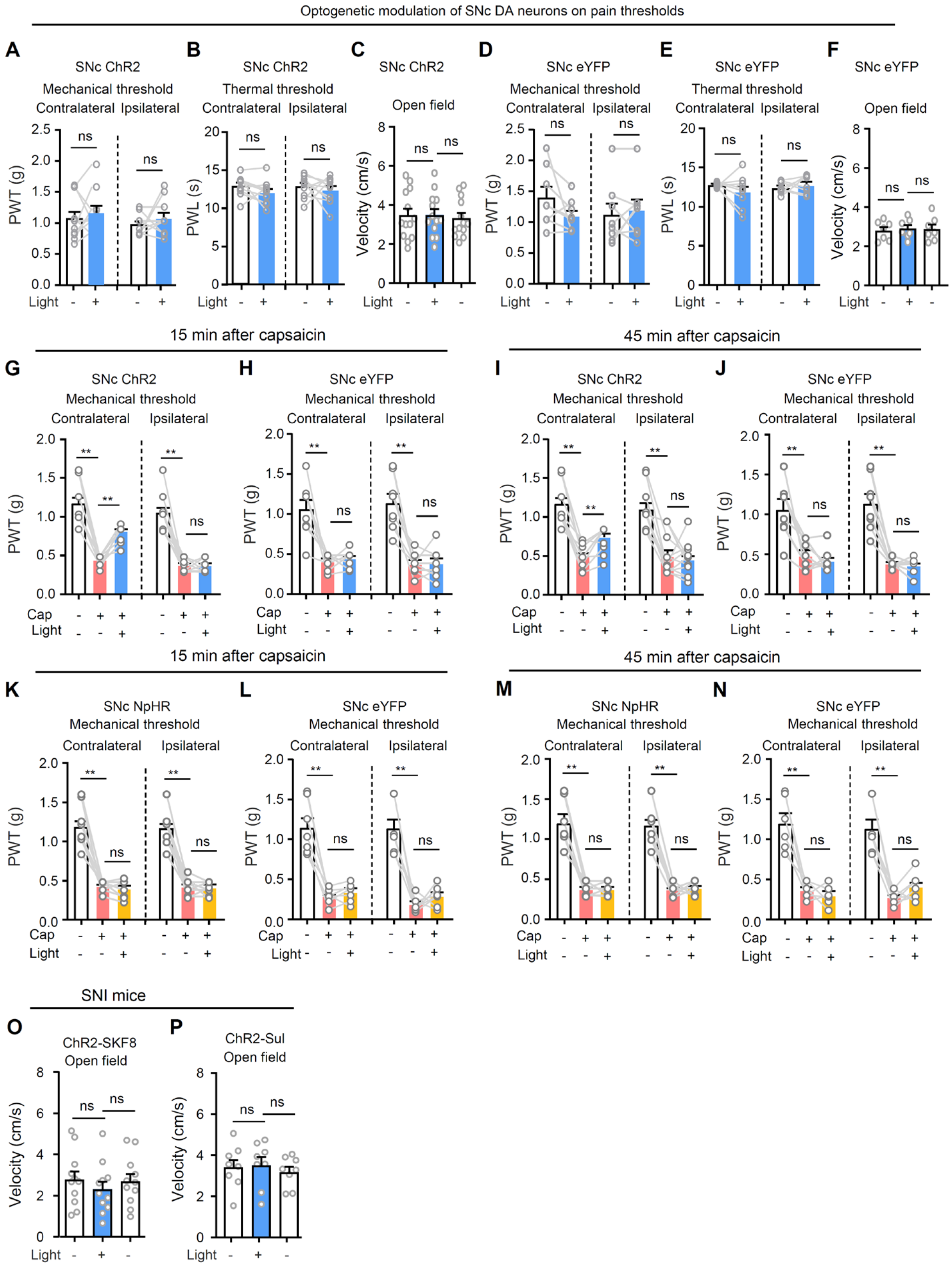

Figure S2 Effects of bidirectional optogenetic modulation of SNc DA neurons on inflammatory pain.

(A-F) PWT, PWL, and velocity of locomotion in ChR2 (n = 10) **(A-C)** and eYFP (n = 8) **(D-F)** mice before and during blue light illumination of the right SNc in naïve mice. **(A)** PWT. Contralateral: $t = 0.81$, $p = 0.44$. Ipsilateral: $t = 0.96$, $p = 0.36$. **(B)** PWL. Contralateral: $t = 1.61$, $p = 0.14$. Ipsilateral: $t = 0.62$, $p = 0.55$. **(C)** Velocity. $F_{(1.150, 13.80)} = 0.53$, $p = 0.50$. **(D)** PWT. Contralateral: $t = 1.79$, $p = 0.12$. Ipsilateral: $t = 0.61$, $p = 0.56$. **(E)** PWL. Contralateral: $t = 1.16$, $p = 0.28$. Ipsilateral: $t = 0.79$, $p = 0.46$. **(F)** Velocity. $F_{(1.373, 8.236)} = 0.21$, $p = 0.73$.

(G-J) PWT in ChR2 (n = 11) and eYFP (n = 7) mice before and during blue light illumination of the SNc in mice before and 15 and 45 min after saline or capsaicin (Cap) injection onto the lower hind leg.

(G) PWT in ChR2 mice. Contralateral: $F_{(1.327, 14.60)} = 53.74$, $p < 0.0001$; $t = 8.57$, $p < 0.0001$, Cap vs Control; $t = 9.61$, $p < 0.0001$, Cap vs Cap+light. Ipsilateral: $F_{(1.234, 12.34)} = 78.40$, $p < 0.0001$; $t = 8.36$, $p < 0.0001$, Cap vs control; $t = 0.08$, $p = 0.99$, Cap vs Cap+light.

(H) PWT in eYFP mice. Contralateral: $F_{(1.188, 8.316)} = 19.13$, $p = 0.0017$; $t = 4.59$, $p = 0.005$, Control vs Cap; $t = 0.75$, $p = 0.73$, Cap vs Cap+light. Ipsilateral: $F_{(1.301, 9.105)} = 26.76$, $p = 0.0003$; $t = 5.53$, $p = 0.002$, Control vs Cap; $t = 0.11$, p$P = 0.99$, Cap vs Cap+light.

(I) PWT in ChR2 mice. Contralateral: $F_{(1.373, 15.10)} = 43.07$, $p < 0.0001$; $t = 8.65$, $p < 0.0001$, Control vs Cap; $t = 5.47$, $p = 0.0004$, Cap vs Cap+light. Ipsilateral: $F_{(1.956, 21.52)} = 28.85$, $p < 0.0001$; $t = 6.22$, $p = 0.0001$, Control vs Cap; $t = 0.51$, $p = 0.85$, Cap vs Cap+light.

(J) PWT in eYFP mice. Contralateral: $F_{(1.426, 9.983)} = 19.97$, $p < 0.0001$; $t = 4.67$, $p = 0.005$, Control vs Cap; $t = 0.95$, $p = 0.61$, Cap vs Cap+light. Ipsi: $F_{(1.120, 7.840)} = 36.38$, $p < 0.0001$; $t = 6.39$, $p = 0.0007$, Control vs Cap; $t = 0.81$, $p = 0.69$, Cap vs Cap+light.

(K-N) PWT in NpHR (n = 9) and eYFP (n = 7) mice before and during yellow light illumination of the SNc in mice before and 15 and 45 min after saline or capsaicin injection onto the lower hind leg.

(K) PWT in NpHR mice. Contralateral: $F_{(1.155, 9.238)} = 57.98$, $p < 0.0001$; $t = 8.20$, $p <$

0.0001, Control vs Cap; $t = 1.01$, $p = 0.56$, Cap vs Cap+light. Ipsilateral: $F_{(1.334, 10.68)} = 78.43$, $p < 0.0001$; $t = 9.39$, $P < 0.0001$, Control vs Cap; $t = 0.30$, $p = 0.95$, Cap vs Cap+light.

(L) PWT in eYFP mice. Contralateral: $F_{(1.132, 6.789)} = 40.12$, $p < 0.0001$; $t = 6.79$, pP = 0.001, Control vs Cap; $t = 1.34$, $p = 0.41$, Cap vs Cap+light. Ipsilateral: $F_{(1.465, 8.792)} = 38.95$, $p < 0.0001$; $t = 6.68$, $p = 0.001$, Control vs Cap; $t = 1.32$, $p = 0.42$, Cap vs Cap+light.

(M) PWT in NpHR mice. Contralateral: $F_{(1.136, 6.814)} = 50.07$, $P < 0.0001$; $t = 7.61$, $P = 0.0005$, Control vs Cap; $t = 0.00$, $p = 1.00$, Cap vs Cap+light. Ipsilateral: $F_{(1.194, 8.358)} = 76.61$, $p < 0.0001$; $t = 11.49$, $p < 0.0001$, Control vs Cap; $t = 11.49$, $p < 0.0001$, Control vs Cap; $t = 0.38$, $p = 0.92$, Cap vs Cap+light.

(N) PWT in eYFP mice. Contralateral: $F_{(1.280, 6.400)} = 23.60$, P = 0.002; $t = 5.34$, $p = 0.008$, Control vs Cap; $t = 0.87$, $p = 0.67$, Cap vs Cap+light. Ipsilateral: $F_{(1.297, 7.783)} = 29.95$, $p < 0.0001$; $t = 6.69$, $p = 0.001$, Control vs Cap; $t = 2.01$, $p = 0.17$, Cap vs Cap+light.

(O, P) Effect of optogenetic stimulation of SNc DA neurons on velocity of locomotion in the open field arena in ChR2 mice subjected to intraperitoneal injection of SKF83566 (SKF8) ($n = 11$) or sulpiride (Sul) ($n = 8$). **(O)** $F_{(1.306, 13.06)} = 2.41$, $p = 0.14$. **(P)** $F_{(1.315, 9.206)} = 0.55$, $p = 0.52$. One-way repeated measures ANOVAs for **(G-P)**.

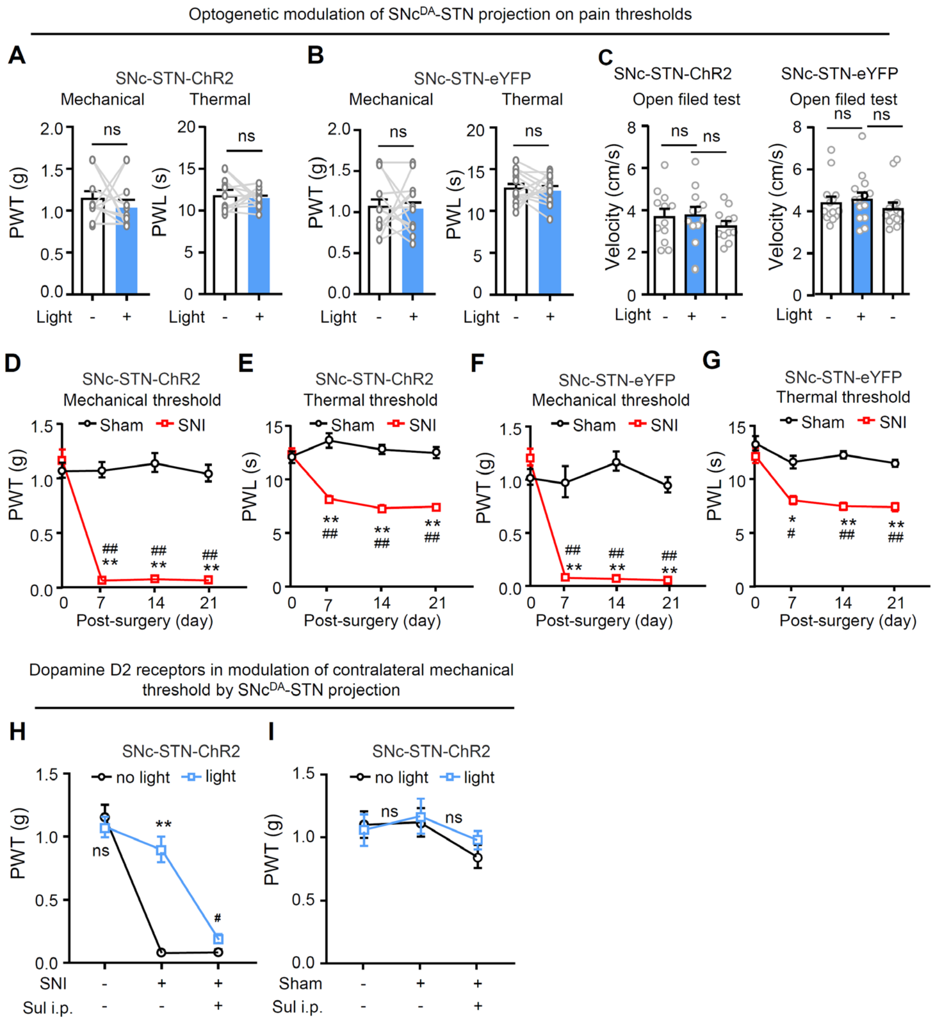

Figure S3 Optogenetic stimulation of the SNc-STN DA projection on pain thresholds.

SNc DA neurons in DAT-Cre mice were transfected with ChR2-eYFP or eYFP by intracranial injection of viral vectors as shown in Figure 3A, and an optical fiber was implanted into the STN (Figure 3D).

(A-C) Effect of blue light illumination of the right STN on PWT and PWL on the left hind paw, and movement velocity in the open field arena in ChR2 and eYFP mice. **(A)** PWT in ChR2 mice: $t = 0.77$, $p = 0.45$; PWL in ChR2 mice: $t = 0.42$, $p = 0.68$. $n = 9$ in ChR2, $n = 9$ in eYFP. **(B)** PWT in eYFP mice: $t = 0.21$, $p = 0.84$; PWL in eYFP mice: $t = 0.50$, $p = 0.62$. $n = 9$ in ChR2, $n = 9$ in eYFP. **(C)** Movement velocity in ChR2 and eYFP mice. SNc-STN-ChR2: $F_{(1.799, 19.79)} = 1.48$, $p = 0.25$. SNc-STN-eYFP: $F_{(1.363, 17.72)} = 1.77$, $p = 0.20$. $n = 12$ in ChR2, $n = 13$ in eYFP. One-way repeated measures ANOVA.

(D-G) PWT and PWL in ChR2 ($n = 11$) and eYFP ($n = 7$) DAT-Cre mice subjected to virus injection into the right SNc, optical fiber implantation into the right STN, and

spared nerve injury (SNI) of the sciatic nerve on the left side. **(D)** PWT in ChR2 mice. Sham: $F_{(2.695, 26.95)} = 0.25$, $p = 0.84$. SNI: $F_{(1.069, 10.69)} = 127.3$, $p < 0.0001$. **(E)** PWL in ChR2 mice. Sham: $F_{(2.073, 20.73)} = 1.90$, $p = 0.17$. SNI: $F_{(2.111, 21.11)} = 51.26$, $p < 0.0001$. **(F)** PWT in eYFP mice. Sham: $F_{(2.150, 12.90)} = 0.88$, $p = 0.44$. SNI: $F_{(1.133, 6.798)} = 215.7$, $p < 0.0001$.**(G)** PWT in eYFP mice. Sham: $F_{(2.156, 12.94)} = 2.65$, $p = 0.11$. SNI: $F_{(1.954, 11.73)} = 22.66$, $p < 0.0001$. * $p < 0.05$, ** $p < 0.01$, SNI vs control; # $p < 0.05$, ## $p < 0.01$, SNI vs Sham.

(H, I) Effects of optogenetic stimulation of the right SNc-STN DA projection on PWT on the left hind paw in ChR2 mice subjected to SNI **(H)** or sham **(I)** surgery on the left side with or without intraperitoneal injection of sulpiride (Sul). **(H)** PWT in SNI. $F_{(2.509, 25.09)} = 56.34$, $p < 0.0001$. q = 1.08, $P = 0.97$, control vs light; q = 11.07, $p = 0.0002$, SNI vs SNI+light; q = 3.22, $p = 0.28$, SUL vs Sul+light. $n = 11$. **(I)** PWT in Sham. $F_{(2.693, 13.47)} = 1.01$, $p = 0.41$. $n = 6$. * $p < 0.05$, ** $p < 0.01$, compared between with and without light.

Two-tailed paired *t*-test for **(A, B)**. One-way repeated measures ANOVAs for **(C-G)**. Two-way repeated measures ANOVAs for **(H, I)**.

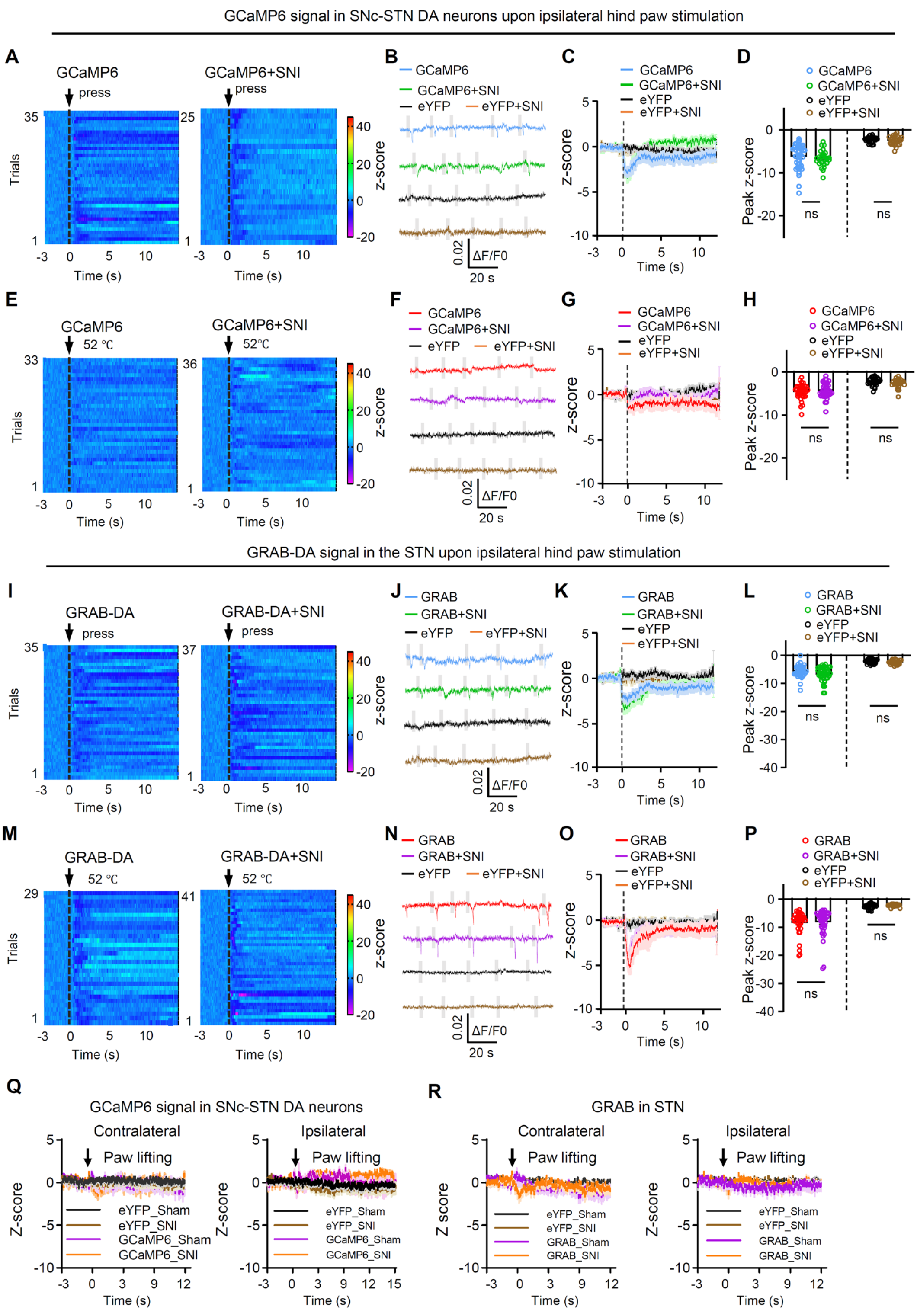

Figure S4 Effects of mechanical and thermal stimulation of the ipsilateral hind paw on SNc-STN DA neurons and STN dopamine level.

(A-D) Heat maps **(A)**, representative traces of raw data **(B)**, average traces **(C)**, and summary of peak z-score **(D)** showing pain responses in SNc-STN DA neurons to mechanical stimulation onto the ipsilateral hind paw in eYFP and GCaMP6 mice

subjected to either sham or SNI surgery. $F_{(3, 120)} = 43.61, p < 0.0001$. $t = 8.43, p < 0.0001$, GCaMP6 (n = 35) vs eYFP (n = 30). $t = 0.61$, $P = 0.96$, GCaMP6 vs GCaMP6+SNI (n = 25). All data are from 6 mice in each group.

(E-H) Heat maps **(E)**, representative traces of raw data **(F)**, average traces **(G)**, and summary of peak z-score **(H)** showing pain responses in SNc-STN DA neurons to thermal stimulation on the ipsilateral hind paw in eYFP and GCaMP6 mice subjected to either sham or SNI surgery. $F_{(3, 123)} = 19.72, p < 0.0001$. $t = 6.15, p < 0.0001$, GCaMP6 (n = 33) vs eYFP (n = 26). $t = 0.84, p = 0.87$, GCaMP6 vs GCaMP6+SNI (n = 36). All data are from 6 mice in each group.

(I-L) Heat maps **(I)**, representative traces of raw data **(J)**, average traces **(K)**, and summary of peak z-score **(L)** showing pain responses in STN dopamine release to mechanical stimulation onto the ipsilateral hind paw in eYFP and GRAB-DA mice subjected to either sham or SNI surgery. $F_{(3, 127)} = 46.58, p < 0.0001$. $t = 6.15, p < 0.0001$, GRAB (n = 35) vs eYFP (n = 27). $t = 0.66, p = 0.94$, GRAB vs GRAB+SNI (n = 37). All data are from 6 mice in each group.

(M-P) Heat maps **(M)**, representative traces of raw data **(N)**, average traces **(O)**, and summary of peak z-score **(P)** showing pain responses in STN dopamine release to thermal stimulation onto the ipsilateral hind paw in eYFP and GRAB-DA mice subjected to either sham or SNI surgery. $F_{(3, 126)} = 31.23, p < 0.0001$. $t = 6.62, p < 0.0001$, GRAB (n = 29) vs eYFP (n = 25). $t = 0.41, p = 0.99$, GRAB vs GRAB+SNI (n = 41). All data are from 6 mice in each group.

(Q, R) Summarized traces showing changes in GCaMP6 / eYFP signal in SNc-STN DA neurons and GRAB / eYFP signal in the STN upon random paw lifting.

One-way ANOVAs with Bonferroni tests for (**D**, **H**, **L**, **P**).

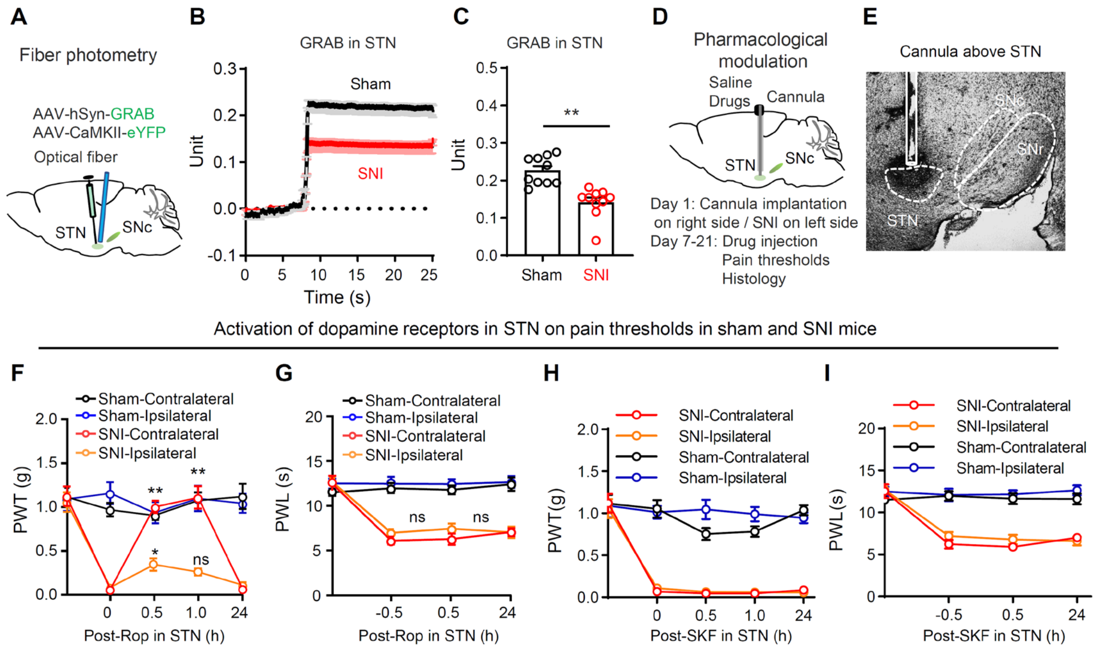

Figure S5 Modulation of pain thresholds by dopamine receptors in the STN.

(A-C) Schematic diagram of virus injection **(A)**, summarized GRAB signal traces **(B)** and quantification **(C)** of fiber photometry recording of GRAB signal in the STN. t = 4.90, p = 0.0001. n = 10 in each group.

(D-I) Schematic diagram **(D)** and representative image **(E)** for mechanical PWT and thermal PWL on either hind paw in sham and SNI mice before and after injection of dopamine receptor agonists.

(F) Effects of ropinirole (Rop) injection into the right STN on PWT on hind paws of sham and SNI mice. Contralateral SNI: $F_{(2.111, 12.67)} = 46.24$, $p < 0.0001$. q = 17.83, $p < 0.0001$, 0.5 h after vs before Rop. q = 1.44, p = 0.84, 0.5 h after Rop vs control. Ipsilateral SNI: $F_{(1.716, 10.30)} = 60.05$, $p < 0.0001$. q = 5.46, p = 0.044, 0.5 h after vs before Rop. q = 15.65, $p < 0.0001$, 0.5 h after rop vs control.

(G) Effects of Rop injection into the right STN on PWL on hind paws of sham and SNI mice. Contralateral SNI: $F_{(2.048, 12.29)} = 21.93$, $p < 0.0001$. q = 0.33, P = 0.99, -0.5 h vs 0.5 h. Ipsilateral SNI: $F_{(2.179, 13.08)} = 31.80$, $p < 0.0001$. q = 1.09, p = 0.86, 0.5 h vs -0.5 h.

(H) Effects of SKF38393 (SKF) injection into the right STN on PWT on hind paws of sham and SNI mice. Contralateral SNI: Group, $F_{(1, 12)} = 110.5$, $p < 0.0001$; Time, $F_{(2.085, 25.03)} = 52.21$, $p < 0.0001$. Ipsilateral SNI: $F_{(1, 12)} = 188.8$, $p < 0.0001$; Time, $F_{(2.197, 26.37)} = 18.97$, $p < 0.0001$.

(I) Effects of SKF38393 (SKF) injection into the right STN on PWL on hind paws of bilateral sham and SNI mice. Contralateral SNI: Group, $F_{(1, 12)} = 84.77$, $p < 0.0001$; Time, $F_{(2.585, 31.02)} = 14.12$, $p < 0.0001$. Ipsilateral SNI: Group, $F_{(1, 12)} = 72.40$, $p < 0.0001$; Time, $F_{(2.355, 28.25)} = 17.32$, $p < 0.0001$.

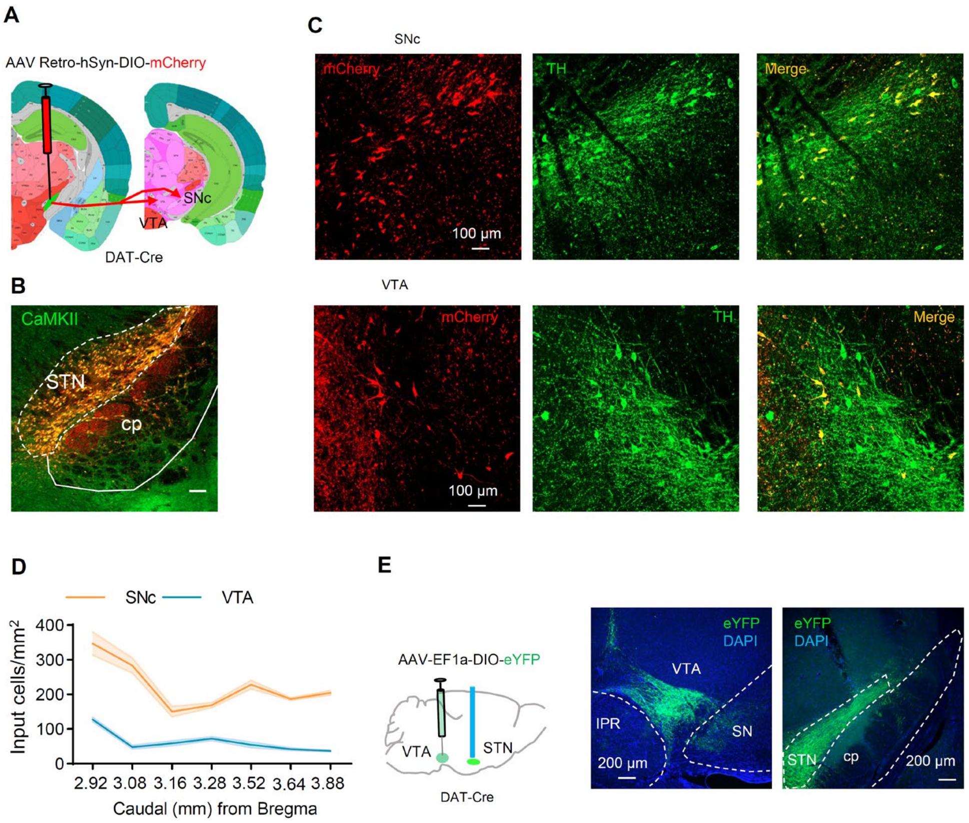

Figure S6 The STN receives dopaminergic inputs from the SNc and ventral tegmental area.

(A) Schematic diagram for virus (AAV retro-hSyn-DIO-mCherry) injection for retrograde tracing of dopaminergic inputs to the STN in DAT-Cre mice.

(B-D) Representative images showing virus injection **(B)** and retrograde labeling (mCherry+) of STN-projecting dopaminergic neurons (TH+) in the SNc and ventral tegmental area (VTA) **(C)** and summary **(D)** of distribution of mCherry-labeled SNc and VTA dopaminergic neurons.

(E, F) Schematic diagram **(E)** for virus (AAV-EF1α-DIO-eYFP) injection into the VTA and representative images **(F)** showing that VTA dopaminergic neurons (green) project to the STN.

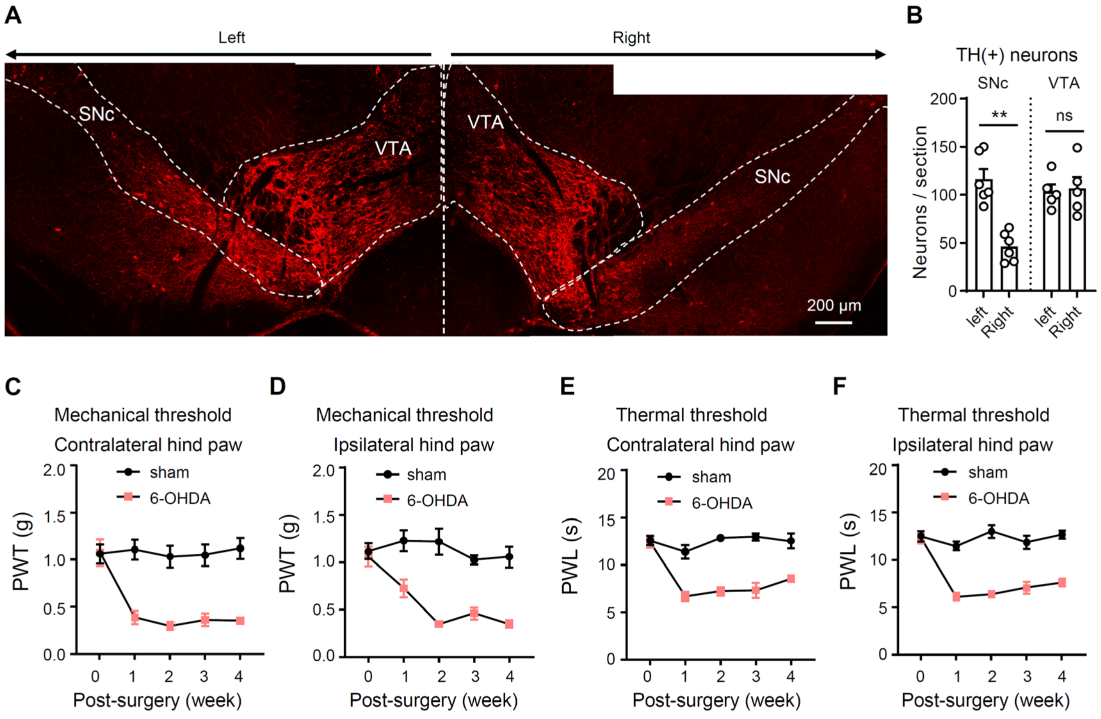

Figure S7 Development of hyperalgesia in parkinsonian mice.

Mice received injection of saline (n = 7) or 6-OHDA (n = 7) into the medial forebrain bundle. Mechanical and thermal thresholds were measured before and 4 weeks after the injection.

(A, B) Representative image **(A)** and summary **(B)** showing 6-OHDA injection into the medial forebrain bundle severely damaged SNc DA neurons (n = 6 mice), but not VTA DA neurons (5 mice). $F_{(1, 9)} = 23.86$, $p = 0.0009$. Two-way ANOVA.

(C, D) PWT on contralateral **(C)** and ipsilateral hind paws **(D)** in mice subjected to saline or 6-OHDA injection into the right medial forebrain bundle. **(C)** Contralateral PWT. Time, $F_{(2.649, 31.79)} = 6.84$, $P = 0.0016$; Group: $F_{(1, 12)} = 51.02$, $p < 0.0001$. **(D)** Ipsilateral PWT. Time, $F_{(2.602, 31.22)} = 7.592$, $p = 0.0009$; Group, $F_{(1, 12)} = 68.88$, $p < 0.0001$. Two-way repeated measures ANOVA.

(E, F) PWL on contralateral **(E)** and ipsilateral hind paws **(F)** in mice subjected to saline or 6-OHDA injection into the right medial forebrain bundle. **(E)** Contralateral PWL. Time, $F_{(3.125, 37.50)} = 10.03$, $p < 0.0001$; Group, $F_{(1, 12)} = 128.1$, $p < 0.0001$. **(F)** Ipsilateral PWL. Time, $F_{(3.249, 38.99)} = 16.68$, $p < 0.0001$; Group, $F_{(1, 12)} = 107.7$, $p < 0.0001$. Two-way repeated measures ANOVA.

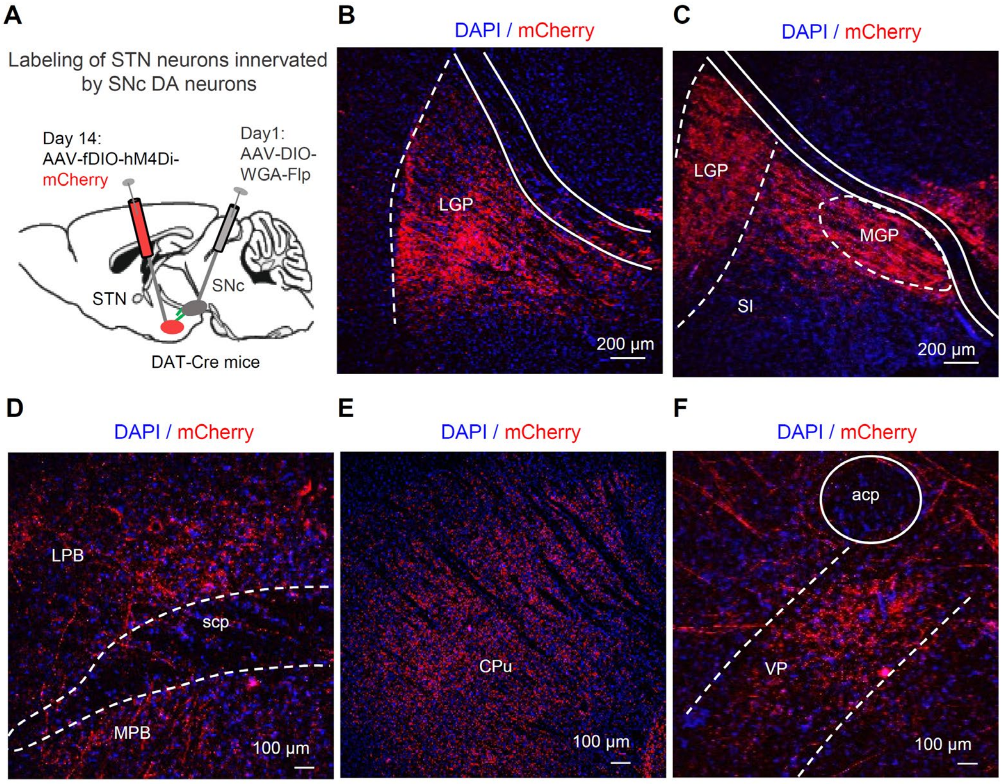

Figure S8 Projections of STN neurons innervated by SNc DA neurons.

Schematic diagram **(A)** for labeling of STN neurons innervated by SNc DA neurons and representative images showing the axonal projections of these neurons to the lateral globus pallidus (LGP) **(B)**, medial globus pallidus (MGP) **(C)**, lateral and medial parabrachial nucleus (LPB, MPB) **(D)**, caudate putamen (CPu) **(E)**, and ventral pallidum (VP) **(F)**. acp, anterior commissure posterior. scp, superior cerebellar peduncle. SI, substantia innominata.

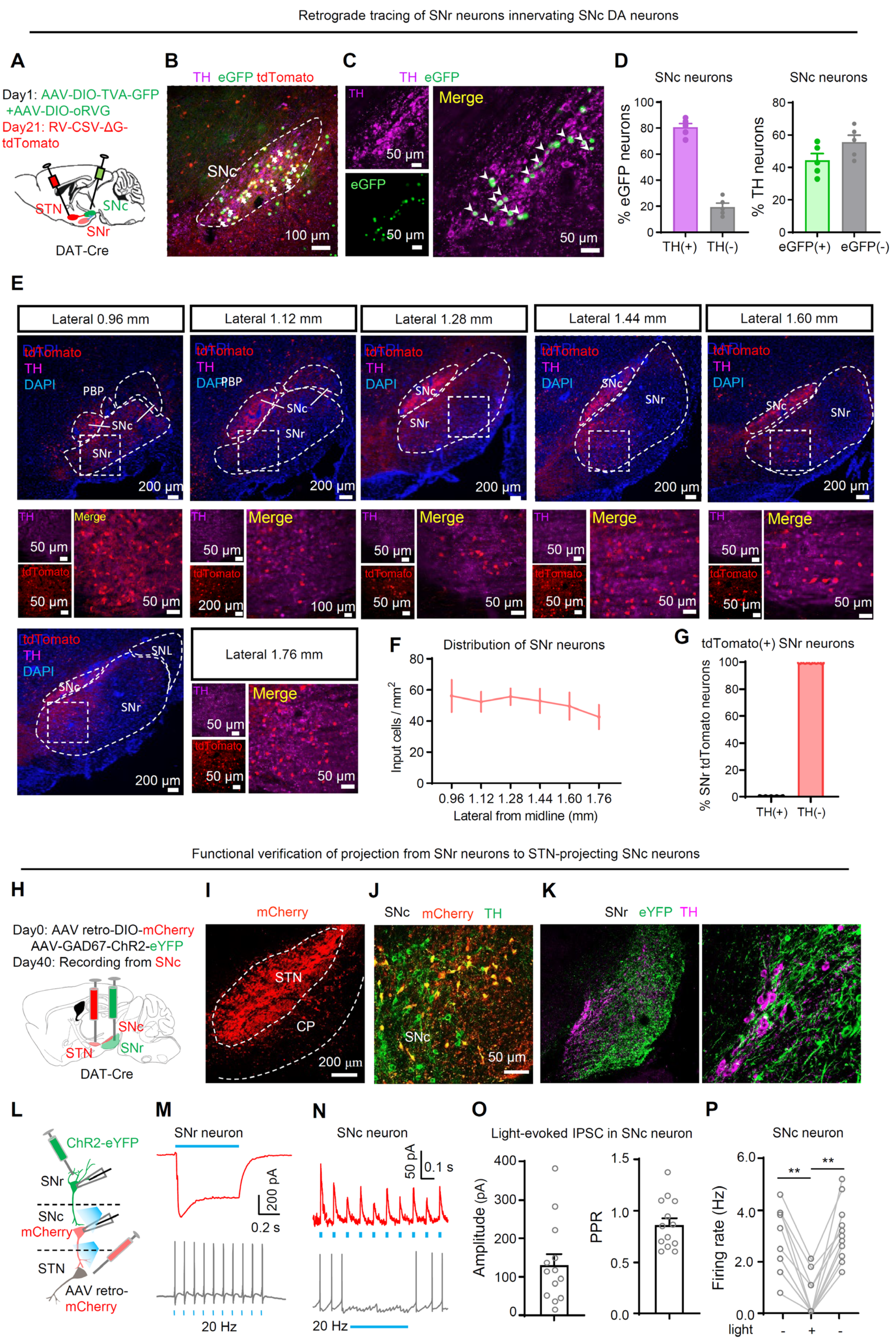

Figure S9 STN-projecting SNc dopaminergic neurons are controlled by GABAergic neurons in the substantia nigra pars reticulata.

(A) Schematic diagram of virus injection for retrograde labeling of upstream nuclei

innervating STN-projecting SNc dopaminergic neurons.

(B-D) Representative images **(B, C)** and summary showing virus-labeled SNc neurons and the specificity **(D**, left panel**)** and efficiency **(D**, right panel**)** of the virus.

(E-G) Representative images **(E)** and summary **(F)** showing the distribution of upstream neurons of SNc-STN dopaminergic neurons in the substantia nigra pars reticulata (SNr). **(G)** TH-antibody-staining of tdTomato-labeled SNr neurons.

(H) Schematic diagram for viral injection to label STN-projecting SNc dopaminergic neurons with mCherry and SNr GABAergic neurons with ChR2-eYFP.

(I) A representative image showing injection site of retrograde virus in the STN.

(J) A representative image showing that retrogradely labeled (mCherry) STN-projecting SNc neurons (red) are TH(+) (green).

(K) A representative image showing that ChR2-eYFP expressing SNr neurons (green) are not TH(+) (purple).

(L) Schematic diagram for virus injection and patch-clamp recordings from SNr and SNc neurons.

(M) Typical traces showing that blue light evoked inward currents (upper trace) (0.6 s, 1 mW) in voltage-clamp mode at the holding potential of -50 mV, and 10 blue light pulses (2 ms, 1 mW, 20 Hz) evoked spikes (lower trace) in current-clamp mode in a ChR2-eYFP-labeled SNr neuron.

(N) Blue light (2 ms, 1 mW, 10 Hz) evoked outward currents (upper trace) in voltage-clamp mode at the holding potential of -45 mV, and inhibited spontaneous firing in SNc DA neurons labeled (mCherry) with retrograde virus injected into the STN.

(O) Summary of amplitude and paired-pulse ratio of blue light-evoked outward currents in in STN-projecting SNc dopaminergic neurons ($n = 13$).

(P) Summary of inhibition of spontaneous firing in STN-projecting SNc DA neurons by activation of SNr GABAergic projection to the SNc. $F_{(1.482, 14.82)} = 53.36, p < 0.0001$; $t = 7.99, P < 0.0001$, baseline vs light. $n = 11$ neurons.

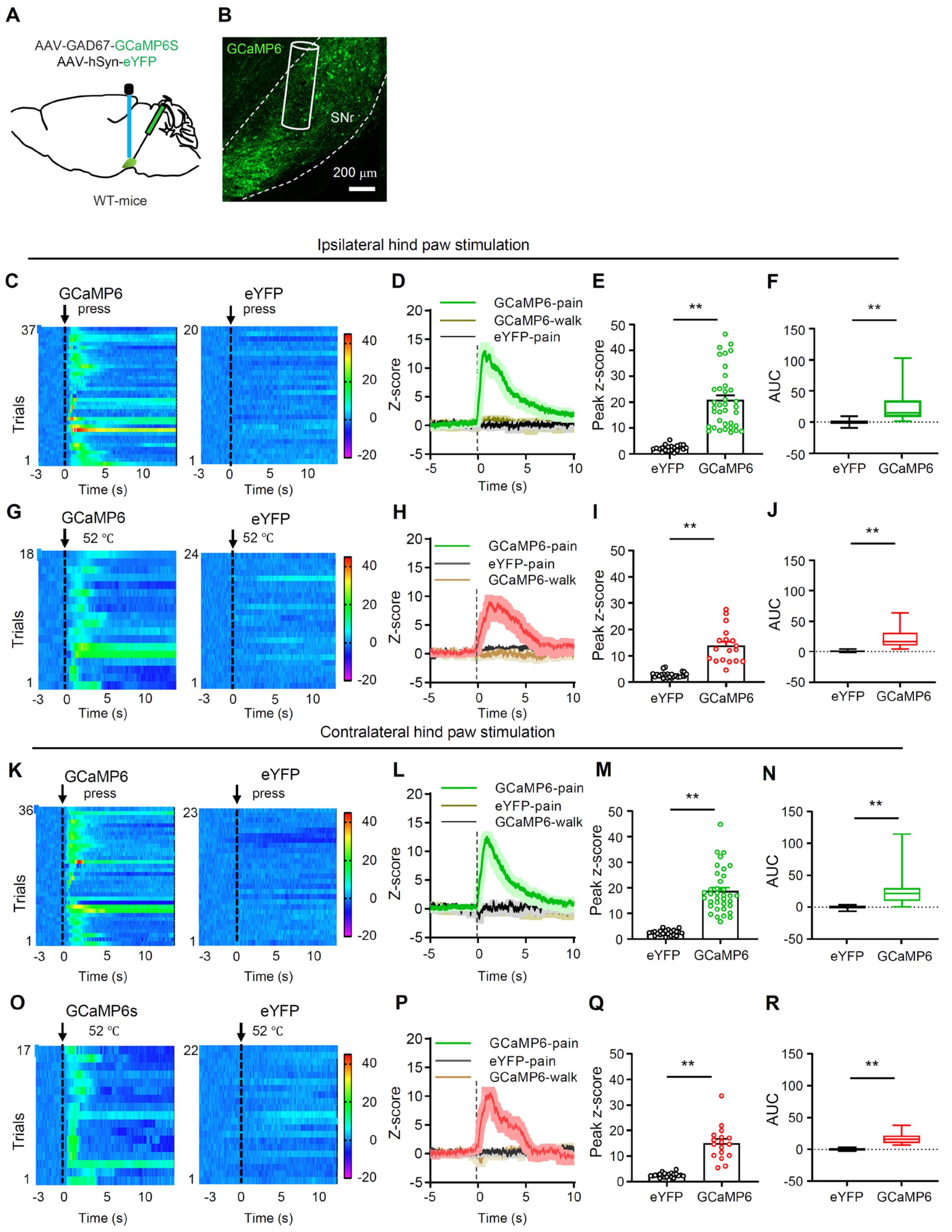

Figure S10 Mechanical and thermal pain stimuli enhance the activity of SNr GABAergic neurons.

(A, B) Schematic diagram **(A)** and a representative image **(B)** for fiber photometry recording of GCaMP6 signal in SNr GABAergic neurons.

(C-F) Heat maps **(C)**, averaged traces **(D)**, summary of peak effect **(E)**, and area under the curve (AUC) **(F)** for the effect of ipsilateral mechanical stimulation on GCaMP6 and eYFP signal in the SNr. Peak: $t = 7.34$, $p < 0.0001$. AUC: $t = 5.08$, $p < 0.0001$. $n =$

20 in eYFP, $n = 37$ in GCaMP6.

(G-J) Heat maps **(G)**, averaged traces **(H)**, summary of peak effect **(I)**, and AUC for the effect **(J)** of ipsilateral thermal stimulation on GCaMP6 and eYFP signal in the SNr. Peak: $t = 9.04$, $p < 0.0001$. AUC: $t = 5.19$, $p < 0.0001$. $n = 24$ in eYFP, $n = 18$ in GCaMP6.

(K-N) Heat maps **(K)**, averaged traces **(L)**, summary of peak effect **(M)**, and AUC for the effect **(N)** of contralateral mechanical stimulation on GCaMP6 and eYFP signal in the SNr. Peak: $t = 7.99$, $p < 0.001$. AUC: $t = 6.65$, $p < 0.0001$. $n = 23$ in eYFP, $n = 36$ in GCaMP6.

(O-R) Heat maps **(O)**, averaged traces **(P)**, summary of peak effect **(Q)**, and AUC for the effect **(R)** of contralateral thermal stimulation on GCaMP6 and eYFP signal in the SNr. Peak: $t = 8.79$, $p < 0.0001$. AUC: $t = 9.37$, $p < 0.0001$. $n = 22$ in eYFP, $n = 17$ GCaMP6.
